# Association between preoperative MRI radiomic features and methylation-defined phenotypes in non-functioning pituitary adenomas

**DOI:** 10.1007/s11102-026-01755-w

**Published:** 2026-08-31

**Authors:** Ilies Djebbara, Morten Winkler Møller, Ivar Yannick Christensen, Bo Halle, Christian Bonde Pedersen, Frantz Rom Poulsen, Jan Saip Aunan-Diop

**Affiliations:** 1 Department of Neurosurgery, Odense University Hospital, J.B. Winsløws Vej 4, Odense C, 5000 Denmark; 2 Clinical Institute and Brain Research Interdisciplinary Guided Excellence (BRIDGE), University of Southern Denmark, Odense, Denmark; 3 Department of Radiology, Odense University Hospital, Odense, Denmark

**Keywords:** NFPA, Radiomics, DNA methylation, MRI, Tumour regrowth

## Abstract

**Purpose:**

DNA methylation profiling identifies clinically relevant subgroups of non-functioning pituitary adenomas (NFPAs), but requires tumour tissue and is unavailable preoperatively. We investigated whether reported outcome-associated methylation-defined NFPA phenotypes are associated with preoperative MRI radiomic features.

**Methods:**

We performed a single-centre retrospective radiogenomic analysis nested within a previously published, outcome-characterised NFPA methylation cohort. Seventy-four patients with preoperative gadolinium-enhanced T1-weighted MRI were included. Tumours were automatically segmented using a fine-tuned U-Net. The primary analysis tested binary discrimination between the outcome-associated low-risk (k1/k2) and high-risk (k3/k4/k5) methylation strata using a prespecified L1-penalised logistic-regression model with leakage-controlled repeated stratified cross-validation, 0.632 + bootstrap optimism correction, and 1000-shuffle whole-pipeline permutation testing.

**Results:**

Seven radiomic features were false-discovery-rate significant and four survived Bonferroni correction, predominantly LoG-filtered first-order intensity features. The prespecified model separated high-risk from low-risk tumours with balanced accuracy 0.69 (95% CI, 0.58–0.80), AUC 0.73 (0.61–0.84), 0.632 + AUC 0.71, and permutation *p* = 0.001. The SF1-restricted analysis remained significant (balanced accuracy 0.73, AUC 0.70, *p* = 0.008), whereas k3-focused analyses were not significant. Clinical sex-lineage models classified at chance.

**Conclusions:**

Preoperative MRI radiomics was associated, at the group level, with a composite methylation-defined risk label previously associated with postoperative regrowth in the same source cohort. The study was clinically outcome-anchored, but the classifier did not use individual regrowth or progression-free survival as its endpoint and should not be interpreted as an independently validated prognostic model. Pooling k3, k4, and k5 does not establish a shared imaging phenotype of aggressiveness. External outcome-linked validation is required before clinical implementation.

**Supplementary information:**

The online version contains supplementary material available at https://doi.org/10.1007/s11102-026-01755-w.

## Introduction

Non-functioning pituitary adenomas (NFPAs) account for approximately one-third of pituitary adenomas [1, 2]. Surgical resection remains the mainstay treatment for symptomatic or enlarging NFPAs, particularly when mass effect, visual field defects, or progressive tumour growth is present [2, 3]. Despite surgery, postoperative tumour regrowth remains a major long-term management challenge [3, 4]. Current risk assessment relies on clinical, radiological, and histopathological variables, including tumour size, invasion, residual tumour, immunohistochemical lineage, and integrated clinicopathological grading systems [1, 2, 5, 6]. Although these factors are clinically useful, they incompletely capture the biological heterogeneity that drives long-term regrowth risk, particularly within histologically similar NFPA subtypes [5, 7, 8].

Genome-wide DNA methylation profiling can reveal biologically meaningful tumour classes beyond conventional histopathology [9]. In pituitary adenomas, methylation signatures are closely related to transcription-factor lineage and may provide information beyond routine histopathological classification [7, 8, 10]. A previously published NFPA study identified five methylation-defined subgroups (k1–k5): four predominantly SF1-lineage subgroups and one TPIT/PIT1-enriched subgroup [7]. Compared with k1/k2, subgroups k3–k5 were associated with higher postsurgical regrowth risk [7], with k3 showing delayed postoperative tumour-volume expansion within SF1-lineage tumours [7].

These previously published findings provide an established, clinically outcome-associated molecular risk framework for NFPAs [7]. However, DNA methylation profiling requires tumour tissue and is generally unavailable during preoperative surgical planning. The present study addresses a distinct translational question: whether MRI radiomic features can serve as non-invasive imaging correlates of these outcome-associated molecular phenotypes. This question is clinically relevant because MRI is routinely acquired before surgery and may contain quantitative information beyond conventional visual assessment [11, 12]. Radiomics provides a quantitative framework for extracting high-dimensional imaging features from routinely acquired medical images [13, 14]. Radiomic analysis enables quantification of image-derived tumour phenotypes and may capture imaging correlates of intra- and inter-tumour heterogeneity [13, 14]. However, reproducible radiomic analysis requires transparent feature extraction, standardised image processing, careful reporting, and appropriate validation [14–17].

In NFPAs, previous radiomic and radiomic-clinical studies have primarily modelled postoperative recurrence or residual-tumour regrowth, supporting the feasibility of quantitative MRI-based risk modelling in this tumour type. These studies targeted individual clinical outcome labels directly, whereas the present study targeted methylation-defined tumour subgroups and the outcome-associated molecular risk grouping described above [12, 18–20].

We hypothesised that the previously defined, outcome-associated methylation-based high- and low-risk strata [7] exhibit distinct radiomic phenotypes on preoperative contrast-enhanced T1-weighted MRI. The primary objective was to determine whether a leakage-controlled radiomic model could discriminate the prespecified high-risk stratum (k3–k5) from the low-risk stratum (k1–k2) [7].

## Methods

### Study population and cohort selection

This was a single-centre retrospective radiogenomic analysis derived from the previously published NFPA methylation cohort reported by Møller et al. [7]. The source cohort comprised 117 adults surgically treated for NFPA at the Department of Neurosurgery, Odense University Hospital, between 2007 and 2017, with tumour tissue available for genome-wide DNA methylation profiling and an assigned k1–k5 methylation subgroup. For the present analysis, eligible patients additionally required a preoperative whole-brain gadolinium-enhanced T1-weighted MRI (MR cerebrum) suitable for automated tumour segmentation and radiomic feature extraction. Dedicated pituitary/sella-protocol MRI alone was not eligible because its restricted field of view, differing in-plane resolution, and sella-centred reconstruction would make radiomic features non-comparable with those from whole-brain acquisitions. DNA methylation subgroup labels were adopted directly from the reference study; no new methylation profiling was performed. The 74-patient radiomic cohort was therefore a nested subset of the outcome-characterised source cohort, not an independent prognostic validation cohort.

**Fig. 1:**
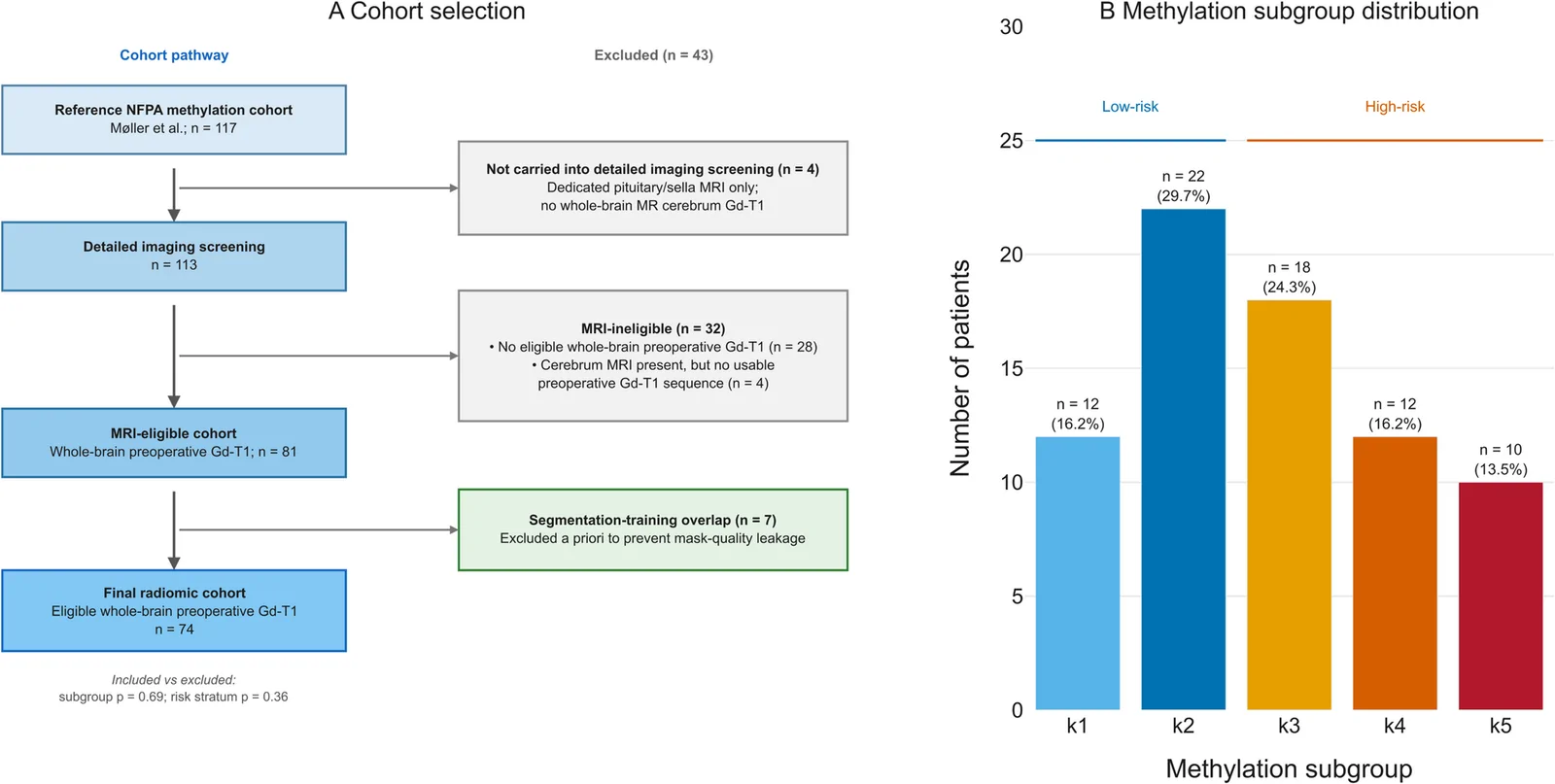
Cohort selection and composition. (**A**) Cohort selection from the reference methylation cohort (*n* = 117) to the final radiomic cohort (*n* = 74). Four patients with only dedicated pituitary/sella MRI and no whole-brain MR cerebrum gadolinium-enhanced T1-weighted sequence were not carried into detailed imaging screening, leaving 113 patients. Detailed imaging screening excluded 32 patients as MRI-ineligible: 28 had no eligible whole-brain preoperative gadolinium-enhanced T1-weighted series, and 4 had a cerebrum MRI but no usable preoperative gadolinium-enhanced T1-weighted sequence. This left 81 MRI-eligible patients. Seven patients overlapping the segmentation-model fine-tuning set were then excluded to prevent mask-quality leakage, yielding the final cohort of 74. Included and excluded patients did not differ in methylation-subgroup distribution (*p* = 0.69) or risk-stratum distribution (*p* = 0.36). (**B**) Distribution of methylation subgroups in the radiomic cohort. Subgroups k1 and k2 were defined as low-risk, whereas k3, k4, and k5 were defined as high-risk according to the reference methylation study. Gd-T1, gadolinium-enhanced T1-weighted MRI; NFPA, non-functioning pituitary adenoma Alt text: Flow diagram showing progression from the 117-patient reference methylation cohort to 113 patients undergoing detailed imaging screening, 81 MRI-eligible patients, and the final 74-patient radiomic cohort. Side boxes show four patients not carried into screening, 32 MRI-ineligible patients, and seven segmentation-training overlaps, alongside a bar chart of the final k1–k5 methylation-subgroup distribution

Of the 117 patients, 4 had only a dedicated pituitary/sella MRI and no whole-brain MR cerebrum gadolinium-enhanced T1-weighted sequence and were not carried into detailed imaging screening, leaving 113 patients. The detailed imaging screen excluded 32 patients as MRI-ineligible: 28 had no eligible whole-brain preoperative gadolinium-enhanced T1-weighted MR cerebrum series in the institutional archive (only pituitary/sella-protocol imaging or no MRI), and 4 had a cerebrum MRI but no usable preoperative gadolinium-enhanced T1-weighted sequence. This left 81 patients with eligible MRI. A further 7 had served as manually annotated segmentation-model fine-tuning cases and were excluded a priori to prevent mask-quality leakage. The final radiomic cohort therefore comprised 74 patients, all analysed from whole-brain MR cerebrum acquisitions using automatically generated masks only (Fig. 1). Baseline characteristics of the final cohort are summarised in Table 1.

**Table 1:** Patient and tumour characteristics of the radiomic cohort. Patient and tumour characteristics are shown for the total radiomic cohort and stratified by methylation-defined low-risk and high-risk groups. Low-risk tumours comprised k1 and k2; high-risk tumours comprised k3, k4, and k5. Data are shown as n (%) unless otherwise specified

Variable	Total (*n* = 74)	Low-risk (*n* = 34)	High-risk (*n* = 40)	*p*
Sex				
Male	47 (63.5%)	25 (73.5%)	22 (55.0%)	
Female	27 (36.5%)	9 (26.5%)	18 (45.0%)	0.146
Immunohistochemical lineage				
SF1	60 (81.1%)	32 (94.1%)	28 (70.0%)	
TPIT	8 (10.8%)	0 (0.0%)	8 (20.0%)	
PIT1	2 (2.7%)	0 (0.0%)	2 (5.0%)	
Not determined	4 (5.4%)	2 (5.9%)	2 (5.0%)	0.020
Automated preoperative tumour volume, cm³				
Median (IQR)	7.3 (4.5–10.3)	7.7 (4.3–9.7)	6.9 (4.5–13.2)	0.927
Invasive growth				
Yes	43 (58.1%)	17 (50.0%)	26 (65.0%)	
No	31 (41.9%)	17 (50.0%)	14 (35.0%)	0.240
PANOMEN-3 grade				
Grade 1	5 (6.8%)	1 (2.9%)	4 (10.0%)	
Grade 2	33 (44.6%)	17 (50.0%)	16 (40.0%)	
Grade 3	36 (48.6%)	16 (47.1%)	20 (50.0%)	0.407
MRI protocol				
3D post-contrast T1-weighted	73 (98.6%)	34 (100.0%)	39 (97.5%)	
2D post-contrast T1-weighted SE	1 (1.4%)	0 (0.0%)	1 (2.5%)	1.000
Methylation subgroup				
k1	12 (16.2%)	12 (35.3%)	—	—
k2	22 (29.7%)	22 (64.7%)	—	—
k3	18 (24.3%)	—	18 (45.0%)	—
k4	12 (16.2%)	—	12 (30.0%)	—
k5	10 (13.5%)	—	10 (25.0%)	—

P-values are descriptive and were not adjusted for multiple testing. Exact tests were used for categorical variables, and Mann–Whitney U test was used for automated preoperative tumour volume. *SE* spin echo, *IQR* interquartile range, *NFPA* non-functioning pituitary adenoma

The primary analysis tested binary discrimination between methylation-defined low-risk tumours (n = 34), comprising k1 and k2, and high-risk tumours (n = 40), comprising k3, k4, and k5. The binary secondary analyses comprised an SF1-restricted high-risk versus low-risk comparison excluding the predominantly non-SF1 k4 subgroup and two exploratory k3-focused comparisons: k3 versus all other subgroups and k3 versus the low-risk subgroups. A separate exploratory five-class k1-k5 analysis was used to assess multiclass subgroup separability. Radiomic models were trained to classify methylation-defined subgroups and the low-risk/high-risk grouping whose association with postoperative regrowth had been established in the same source cohort. Individual regrowth status, time to regrowth, and progression-free survival were not used as endpoints in the present radiomic models. Accordingly, the study was clinically outcome-anchored but did not directly model outcome. Throughout this manuscript, ‘risk’ denotes the grouping assigned by the reference methylation study and not an individual probability of postoperative regrowth estimated by the present model.

### MRI data and methylation subgroup assignment

Preoperative MRI comprised whole-brain post-contrast T1-weighted MR cerebrum acquisitions obtained for neuronavigation across regional hospital sites; dedicated pituitary/sella-protocol acquisitions were not included. All examinations were acquired on Philips systems. Sixty-seven examinations (90.5%) were acquired at 1.5 T and seven (9.5%) at 3 T. Seventy-one examinations (95.9%) used the dominant 3D post-contrast T1-weighted fast-field-echo protocol; three used minority protocols, including one 2D spin-echo acquisition. Complete repetition time, echo time, flip angle, matrix, in-plane resolution and native slice-thickness ranges are reported in Supplementary Table S1. Before feature extraction, images and masks were resampled to a 1-mm isotropic grid, image intensities were z-score normalised within the non-zero foreground, and radiomic intensities were discretised using a fixed bin count of 32. No scanner-, field-strength- or sequence-specific statistical batch harmonisation, such as ComBat, was applied. These preprocessing steps standardised spatial geometry and intensity scale but cannot eliminate acquisition-dependent radiomic effects.

Contrast-enhanced T1-weighted imaging was selected a priori because the whole-brain post-contrast T1-weighted acquisition used for neuronavigation was the routinely obtained preoperative sequence most directly embedded in the surgical workflow represented by this cohort and available to the treating neurosurgical team during operative planning. The objective was not to identify the biologically optimal MRI contrast, but to determine whether methylation-defined risk information could be recovered from an examination already acquired in routine care. Although T2-weighted and other multiparametric sequences may provide complementary information, they were outside the deliberately pragmatic, workflow-aligned scope of this study.

Methylation subgroup assignment was performed in the reference molecular study using archived FFPE tumour tissue profiled on the Illumina MethylationEPIC v1 array. The five previously defined methylation subgroups were used as originally assigned. Subgroups k1 and k2 were classified as low-risk, whereas k3, k4, and k5 were classified as high-risk based on their previously reported association with increased postsurgical regrowth risk. For the SF1-restricted analysis, k4 was excluded because this subgroup is predominantly TPIT/PIT1-enriched rather than SF1-lineage.

### Manual annotation and automated tumour segmentation

Manual tumour annotations were available for 20 NFPA cases and served as reference masks for segmentation fine-tuning and internal validation. Tumours were delineated on the post-contrast T1-weighted sequence used for radiomic analysis by a trained oncoradiologist with > 10 years of experience in pituitary MRI interpretation, using 3D Slicer version 5.10.0 [21].

A segmentation model that has already seen a given tumour during training will delineate that tumour unrealistically well, which would inflate downstream radiomic performance; segmentation was therefore developed and applied in two strictly separated phases. A five-model ensemble based on the previously published two-dimensional U-Net architecture and pretrained weights was fine-tuned using these 20 manually annotated cases [22]. In Phase 1, five-fold held-out cross-validation was used to estimate segmentation performance, ensuring that each annotated case was evaluated exactly once by a model not trained on that case. In Phase 2, deployment models were fine-tuned on all 20 annotated cases and applied as an ensemble to generate tumour masks for the radiomic cohort. Seven of the 20 fine-tuning cases belonged to the 117-patient source cohort and were excluded a priori from the radiomic cohort; the remaining 13 cases were outside the source cohort. This prevented mask-quality leakage and ensured that all 74 radiomic analyses used automatically generated masks only. Individual DSC estimates were not available for the 74 radiomic cohort cases because manual reference masks were not available for these patients. Detailed model architecture, training parameters, augmentation procedures, and deployment settings are provided in the Supplementary Methods and Supplementary Table S2.

Before radiomic feature extraction, each automatically generated mask underwent automated technical quality control. Recorded measures included foreground voxel count, physical mask volume, bounding-box extent, centroid location and voxel spacing. Empty masks and very small masks containing fewer than 50 voxels were prespecified for exclusion, whereas masks containing 50–199 voxels were flagged as small. These checks were intended to identify gross processing failures rather than establish anatomical accuracy. Manual reference segmentations and systematic visual adjudication were not available for the 74 radiomic-cohort cases, and the automatically generated masks were not manually corrected.

### Radiomic feature extraction and feature reduction

To prevent information leakage, feature-level testing and predictive modelling were separated. We extracted 386 radiomic features per case. For cohort-level univariable testing, redundancy was reduced by Spearman correlation clustering (|r| ≥ 0.90). In the four binary models, standardisation and correlation clustering were refitted within each training fold; L1 regularisation provided embedded feature selection, with the penalty tuned on training data only. No univariable p-value filter was used. Re-extraction with pinned PyRadiomics 3.1.0 using identical settings reproduced all features with ICC ≥ 0.90 (median ICC and Spearman correlation approximately 1.00) and left the optimism-corrected primary AUC essentially unchanged (Supplementary Material).

### Statistical testing and machine-learning classification

When many features are tested against a small cohort, some will appear statistically significant by chance alone; all univariable testing was therefore corrected for the number of comparisons made. Univariable group differences in retained radiomic features were assessed using Kruskal–Wallis testing, with effect sizes estimated as η² and uncertainty quantified using bootstrap 95% confidence intervals. Multiple testing was controlled using Benjamini–Hochberg false discovery rate correction, with q < 0.05 considered statistically significant; Bonferroni correction was reported as a stricter secondary criterion.

Overfitting was addressed through a fixed analysis plan, 0.632 + bootstrap optimism correction, and whole-pipeline permutation testing, which compared observed performance with that obtained after random reassignment of subgroup labels.

Four binary classification analyses were evaluated under the locked, leakage-controlled framework: (1) the primary high-risk versus low-risk comparison, (2) the SF1-restricted high-risk versus low-risk comparison, (3) k3 versus all other subgroups, and (4) k3 versus the low-risk subgroups. A single cross-validation design—repeated stratified five-fold cross-validation with three repeats, corresponding to 15 outer-fold evaluations—was locked and used for both reported performance and permutation testing.

Within each outer training fold, features were standardised and reduced by label-independent Spearman correlation clustering. The prespecified binary classifier was an L1-penalised logistic-regression model, with the regularisation parameter selected by three-fold inner cross-validation. Balanced accuracy was the primary performance metric, and AUC was also reported. Performance estimates were calculated from averaged out-of-fold probabilities and presented with bootstrap 95% confidence intervals. Optimism was assessed using the 0.632 + bootstrap, and calibration was evaluated using the calibration slope, calibration intercept and Brier score.

Statistical significance was assessed using whole-pipeline permutation testing with 1,000 label shuffles. For each permutation, preprocessing, hyperparameter tuning, model fitting and repeated cross-validation were rerun under the same locked framework. The empirical p value was calculated as the proportion of permuted mean balanced-accuracy values that equalled or exceeded the observed value, with + 1 smoothing.

To evaluate whether the primary result was driven by minority acquisition protocols, we performed a post hoc sensitivity analysis that excluded the three minority-protocol examinations and reran the complete prespecified L1-penalised logistic-regression pipeline in the remaining 71 patients. The same repeated five-fold cross-validation, in-fold preprocessing and regularisation tuning, bootstrap confidence intervals, 0.632 + optimism correction, and 1,000-shuffle whole-pipeline permutation test were used. The association between acquisition-protocol group (dominant versus minority) and methylation-risk stratum was assessed with a two-sided Fisher exact test.

A secondary six-family classifier-selection procedure evaluated L1-penalised logistic regression, random forest, calibrated linear support vector machine, shrinkage linear discriminant analysis, CatBoost and XGBoost. Classifier selection was performed by three-fold inner cross-validation within each outer training fold. For the primary and SF1-restricted analyses, the complete classifier-selection procedure was also repeated within each of 1,000 label permutations. A clinical baseline using only sex and immunohistochemical lineage was evaluated under the same outer cross-validation design.

A separate exploratory five-class k1–k5 analysis was conducted using CatBoost and 500-shuffle permutation testing. This multiclass analysis was not rerun under the locked binary-classification framework and is therefore reported separately rather than interpreted as methodologically equivalent to the four binary analyses.

#### Software and ethics

Analyses were performed in Python 3.12.3 on Ubuntu Linux. Deep learning used TensorFlow 2.21.0 with tf_keras 2.21.0; radiomic feature extraction used PyRadiomics, SimpleITK, and nibabel; statistical analyses and machine-learning models used scikit-learn, SciPy, statsmodels, CatBoost, and XGBoost. The full computational environment is provided in the Supplementary Methods.

Ethical approval for the source cohort was granted by the Danish Health Research Ethics Committee system (Acadre 17/46106; Project-ID: S-20170216). Approval for data extraction and handling was granted by the Danish Data Protection Agency (journal no. 16/25477). The present retrospective radiomic analysis was conducted under the same ethical and data-protection approvals as the source cohort. The study was conducted in accordance with the Declaration of Helsinki.

## Results

### Cohort overview

Of the 117 patients in the reference methylation cohort, 4 were not carried into detailed imaging screening because only dedicated pituitary/sella MRI was available, leaving 113 patients. Detailed imaging screening excluded 32 patients as MRI-ineligible: 28 had no eligible whole-brain preoperative gadolinium-enhanced T1-weighted MR cerebrum series and 4 had a cerebrum MRI but no usable preoperative gadolinium-enhanced T1-weighted sequence. This left 81 MRI-eligible patients, of whom 7 were excluded because they overlapped the segmentation-model fine-tuning set, yielding a final radiomic cohort of 74 patients (Fig. 1). The final cohort comprised k1, *n* = 12 (16.2%); k2, *n* = 22 (29.7%); k3, *n* = 18 (24.3%); k4, *n* = 12 (16.2%); and k5, *n* = 10 (13.5%). The low-risk stratum (k1 + k2) included 34 patients (45.9%), and the high-risk stratum (k3 + k4 + k5) included 40 (54.1%). To evaluate selection by methylation class, the 74 included patients were compared with all 43 patients excluded from the 117-patient source cohort. The groups did not differ significantly in methylation-subgroup distribution (χ² = 2.24, df = 4, *p* = 0.69) or risk-stratum distribution (included low/high, 34/40; excluded low/high, 16/27; χ² = 0.85, df = 1, *p* = 0.36). Thus, all five subgroups remained represented and there was no evidence that radiomic eligibility preferentially selected a methylation subgroup or risk stratum. Baseline characteristics of the final cohort are presented in Table 1.

### Automated tumour segmentation performance

Internal held-out cross-validation on the 20 manually annotated cases yielded a median volumetric DSC of 0.867 and mean DSC of 0.808 (SD 0.137; IQR 0.800–0.887). Seventeen cases (85%) achieved DSC ≥ 0.70, 15 (75%) achieved DSC ≥ 0.80 and three (15%) achieved DSC ≥ 0.90. Median absolute volume error was 14.4% and mean absolute volume error was 26.0%. Two cases had DSC < 0.60, reflecting one under-segmentation failure and one over-segmentation failure; case-level metrics are provided in Supplementary Table S4. These results represent internal held-out cross-validation performance and do not establish segmentation performance in an independent external cohort.

All 74 radiomic-cohort masks passed automated technical quality control. All masks were non-empty and had voxel spacing within the predefined tolerance of 1-mm isotropic resolution; none met the very-small (< 50 voxels) or small (50–199 voxels) flag definitions. Mask volumes ranged from 306 to 18,762 mm³ (median, 7,261 mm³), and no case was excluded by the automated mask-quality criteria.

### Radiomic feature extraction and reduction

Radiomic features were extracted from automatically segmented tumour volumes for all 74 included patients. The initial feature set comprised 386 features per case. For cohort-level univariable testing, Spearman correlation clustering retained 164 representative features for the primary binary, five-class, SF1-restricted and k3-versus-rest comparisons and 165 features for the k3-versus-low-risk comparison. These retained feature sets were used for the feature-level statistical analyses. In the four predictive binary analyses, standardisation and correlation reduction were instead refitted independently within each training fold.

### Primary binary high-risk versus low-risk classification

**Fig. 2:**
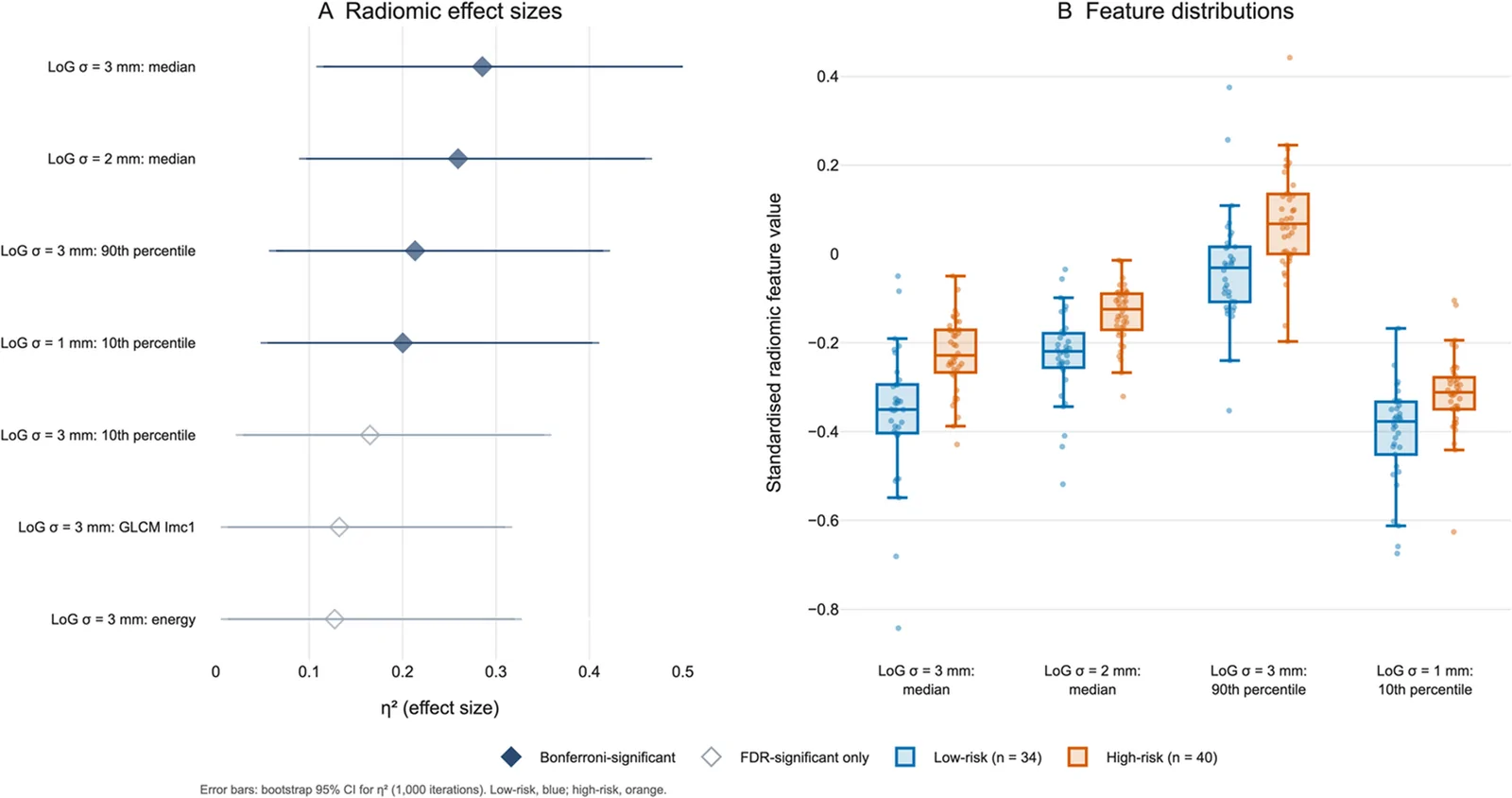
Primary radiomic signal for methylation-defined low-risk versus high-risk NFPA. (**A**) Effect sizes for the seven FDR-significant radiomic features in the primary binary high-risk versus low-risk analysis. Points indicate η² effect sizes from Kruskal–Wallis testing, and horizontal lines indicate bootstrap 95% confidence intervals. Filled diamonds indicate features that remained significant after Bonferroni correction; open diamonds indicate features significant after FDR correction only. (**B**) Distributions of the four Bonferroni-significant features in low-risk and high-risk tumours. Boxplots show median and interquartile range, with individual data points overlaid. Low-risk tumours comprised k1 and k2; high-risk tumours comprised k3, k4, and k5. Full PyRadiomics feature names and complete feature-level statistics are provided in Supplementary Tables S5 and S6. FDR, false discovery rate; LoG, Laplacian of Gaussian; NFPA, non-functioning pituitary adenoma. Alt text: Effect-size plot and boxplots showing radiomic features that differed between methylation-defined low-risk and high-risk tumours

The primary analysis compared high-risk tumours (k3 + k4 + k5; *n* = 40) with low-risk tumours (k1 + k2; *n* = 34). In exploratory univariable testing, seven of 164 features (4.3%) were FDR-significant and four (2.4%) survived Bonferroni correction, all LoG-filtered first-order intensity features (Fig. 2; Supplementary Tables S5–S6).

**Fig. 3:**
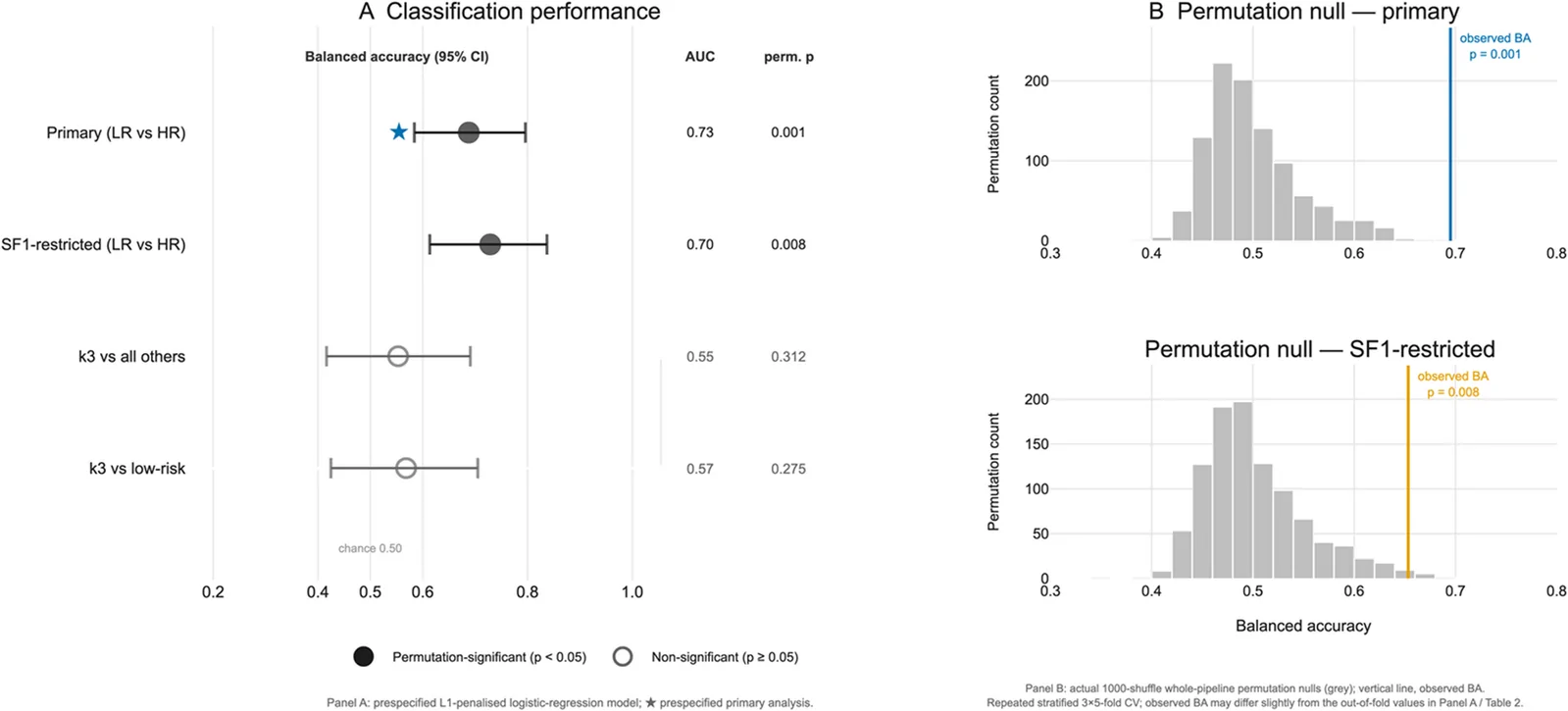
Classification performance and permutation validation across radiomic analyses. (**A**) Balanced accuracy (points) with 95% confidence intervals (horizontal lines) for the radiomic classification analyses shown in Table 2, with AUC annotated for each analysis. Filled circles indicate analyses significant by pipeline-level permutation testing at *p* < 0.05; open circles indicate non-significant analyses. The blue star marks the prespecified primary analysis. Vertical grey ticks mark the chance level for each analysis (0.50 for binary analyses; 0.20 for the five-class analysis). (**B**) Permutation null distributions for the key analyses: primary binary low-risk versus high-risk, SF1-restricted low-risk versus high-risk, and five-class k1–k5 classification. For the binary analyses, null distributions are the 1000-shuffle whole-pipeline permutation nulls of the prespecified model; for the five-class analysis, which was not re-run under the prespecified leakage-controlled pipeline, the null is shown as a normal approximation of the original best-classifier result (CatBoost, 500 shuffles). Vertical lines indicate the observed balanced-accuracy statistic from the permutation-test framework, which used a single stratified 5-fold cross-validation and may therefore differ from the repeated cross-validation and out-of-fold estimates shown in Panel A and Table 2. Permutation p-values are shown for each analysis. LR, low-risk; HR, high-risk; CV, cross-validation; AUC, area under the receiver operating characteristic curve Alt text: Classification performance plot and permutation-test distributions showing that the primary binary, five-class, and SF1-restricted analyses were statistically significant

Under the prespecified leakage-controlled estimator, the L1-penalised logistic-regression model separated high-risk from low-risk tumours with balanced accuracy 0.69 (95% CI, 0.58–0.80) and AUC 0.73 (0.61–0.84). The 0.632 + optimism-corrected AUC was 0.71, compared with an apparent in-sample AUC of 0.90. Whole-pipeline permutation testing with 1000 label shuffles was significant (*p* = 0.001). When the full six-family classifier-selection procedure was permuted in its entirety, the primary analysis remained significant (balanced accuracy 0.73; *p* = 0.001), indicating that the signal was not explained by post hoc classifier selection (Fig. 3; Table 2).

**Table 2:** Summary of the four locked binary analyses and the separate exploratory five-class analysis. The four binary analyses were evaluated using the locked repeated cross-validation and whole-pipeline permutation framework. The five-class k1–k5 analysis was a separate exploratory CatBoost analysis and is shown for completeness

Analysis	*n*	Primary model	Balanced accuracy [95% CI]	AUC [95% CI]	Permutation *p*	0.632 + AUC	Best-of-6 BA/perm *p*
Primary binary (LR vs. HR)	74	L1-logistic	0.69 [0.58–0.80]	0.73 [0.61–0.84]	0.001	0.71	0.73/0.001
Five-class (k1–k5)	74	CatBoost†	0.355†	0.642†	0.002†	—	—
SF1-restricted binary (LR vs. HR)	62	L1-logistic	0.73 [0.61–0.84]	0.70 [0.55–0.83]	0.008	0.62	0.59/0.038
k3 vs. all others	74	L1-logistic	0.55 [0.42–0.69]	0.55 [0.38–0.72]	0.31	0.60	0.52/—
k3 vs. low-risk	52	L1-logistic	0.57 [0.42–0.71]	0.57 [0.41–0.73]	0.27	0.62	0.57/—

The bold row indicates the prespecified primary analysis. The four binary analyses were evaluated using an L1-penalised logistic-regression model under a locked repeated stratified three-times-five-fold cross-validation framework. Balanced accuracy and AUC are shown with bootstrap 95% confidence intervals, and 0.632 + AUC is the optimism-corrected estimate. Binary-analysis permutation p values are based on 1,000 label shuffles of the complete analytical pipeline. The best-of-six column reports the balanced accuracy and permutation p value of the six-family classifier-selection procedure; complete procedure-level permutation testing was performed for the primary and SF1-restricted analyses. †The five-class analysis was a separate exploratory CatBoost analysis using 500-shuffle permutation testing and was not rerun under the locked binary-classification framework; no 0.632 + or best-of-six estimate was calculated. *LR* low-risk, *HR* high-risk, *CI* confidence interval

A clinical baseline using only sex and immunohistochemical lineage classified at chance (AUC 0.52), supporting that the radiomic signal was not simply a lineage surrogate. The effective events-per-variable was low relative to the candidate feature space; absolute performance estimates should therefore be interpreted as internally validated and hypothesis-generating.

### MRI acquisition sensitivity analysis

All three minority-protocol examinations occurred in the high-risk stratum, but protocol group was not significantly associated with methylation-risk stratum (two-sided Fisher exact *p* = 0.245). After excluding these examinations, the complete primary pipeline in the dominant-protocol subset (*n* = 71; 37 high-risk and 34 low-risk) yielded balanced accuracy 0.67 (95% CI, 0.56–0.78), AUC 0.68 (0.55–0.81), 0.632 + AUC 0.68, and a significant whole-pipeline permutation result (*p* = 0.003). Performance was modestly attenuated relative to the full cohort but remained directionally consistent, indicating that the primary signal was not solely attributable to the three minority protocols.

### Exploratory five-class methylation subgroup classification

In the five-class k1-k5 analysis, three of 164 retained features (1.8%) were significant after FDR correction, and all three also survived Bonferroni correction. These were LoG-filtered first-order intensity features, including σ = 2.0–3.0 mm median and σ = 3.0 mm 90th-percentile features. In this separate exploratory analysis, CatBoost achieved balanced accuracy 0.355, macro-F1 0.407 and one-versus-rest AUC 0.642. Performance exceeded the five-class chance level of 0.20, with an empirical permutation p value of 0.002 based on 500 label shuffles. Absolute performance was nevertheless modest, consistent with the small individual subgroup sample sizes, particularly k5 (*n* = 10). Because this multiclass analysis was not rerun under the locked binary-analysis framework, its performance and permutation result should not be directly compared with those of the four binary analyses.

### Sf1-restricted high-risk versus low-risk analysis

The SF1-restricted analysis compared SF1 low-risk tumours (k1 + k2; *n* = 34) with SF1 high-risk tumours (k3 + k5; *n* = 28). Four of 164 features (2.4%) were significant after FDR correction, of which three (1.8%) survived Bonferroni correction. The significant features again predominantly comprised LoG-filtered first-order intensity features, including σ = 3.0 mm median, σ = 2.0 mm median, and σ = 1.0 mm 10th percentile.

In the prespecified SF1-restricted secondary analysis, the comparison of k1/k2 versus k3/k5 tumours (*n* = 62; 28 high-risk events) yielded balanced accuracy 0.73 (95% CI, 0.61–0.84) and AUC 0.70 (0.55–0.83) for the L1-penalised logistic-regression model. Whole-pipeline permutation testing was significant (*p* = 0.008), and the 0.632 + optimism-corrected AUC was 0.62. The secondary six-family classifier-selection procedure was weaker but remained significant (permutation *p* = 0.038). Because k4 was excluded and the sex-lineage clinical baseline classified at chance within SF1 tumours (AUC 0.35), this analysis supports that the primary radiomic signal was not driven solely by lineage composition, although the small number of events makes the result supportive rather than definitive.

### K3-focused exploratory analyses

In the k3-versus-all-others analysis, the prespecified L1-penalised logistic-regression model achieved balanced accuracy 0.55 (95% CI, 0.42–0.69) and AUC 0.55 (0.38–0.72), without significant whole-pipeline permutation evidence (*p* = 0.31). No radiomic feature was FDR-significant.

In the k3-versus-low-risk analysis, the prespecified model achieved balanced accuracy 0.57 (95% CI, 0.42–0.71) and AUC 0.57 (0.41–0.73), again without significant permutation evidence (*p* = 0.27). No radiomic feature was FDR-significant. Thus, neither k3-focused comparison supported a distinct conventional T1-weighted MRI radiomic phenotype for k3 in this cohort.

## Discussion

Methylation-defined NFPA risk strata were associated, at the group level, with the preoperative MRI radiomic phenotype. The strongest signal separated high- from low-risk tumours and persisted after excluding the predominantly non-SF1 k4 subgroup, whereas k3-specific separation was not achieved. Routine contrast-enhanced T1-weighted MRI may therefore capture broader molecular risk biology more readily than individual subgroup granularity, although external validation is required.

The present findings fit with the movement toward biologically refined pituitary adenoma classification. The 2022 WHO classification emphasises tumour lineage, transcription-factor expression, and histopathological subtype as central elements of pituitary adenoma diagnosis [1, 10]. Integrated clinical frameworks such as PANOMEN-3 further highlight the need to combine clinical, radiological, pathological, and molecular information for pituitary tumour prognostication [5, 6]. Molecular and single-cell studies similarly indicate that pituitary adenomas contain biologically distinct subgroups that are not fully resolved by conventional morphology alone [8, 23, 24]. Against this background, the current findings suggest that radiomics may provide a preoperative imaging-level correlate of methylation-defined risk strata, rather than an alternative to molecular classification.

The most consistently significant radiomic features were LoG-filtered first-order intensity features, particularly at σ = 2.0–3.0 mm (Fig. 2), indicating that the discriminative signal was carried primarily by intermediate-scale intensity variation within the enhancing tumour volume rather than by gross tumour size or shape. In practical terms, these features may reflect spatial variation in enhancement, tissue composition, vascularity, cystic or haemorrhagic change, or other architectural heterogeneity; however, these associations were not tested against histopathology in the present cohort (which was unavailable) and remain hypotheses.

The SF1-restricted analysis addressed whether the primary signal merely reflected inclusion of the predominantly TPIT/PIT1-enriched k4 subgroup. After excluding k4, the SF1-restricted comparison remained significant for the prespecified model, and a clinical sex+lineage baseline classified at chance (AUC 0.35 within SF1 tumours; 0.52 overall), supporting the interpretation that the radiomic signal is not explained by lineage. The six-family selection procedure was weaker for SF1 (permutation *p* = 0.038), so this comparison is supportive rather than definitive.

The prespecified primary endpoint reproduced the low-risk/high-risk grouping established in the reference methylation study, but the high-risk class pooled molecularly distinct k3, k4, and k5 tumours. Binary discrimination therefore cannot be interpreted as evidence of one radiomic phenotype of aggressiveness shared by all three subgroups. The observed signal could partly reflect subgroup-specific imaging characteristics or the composition of the pooled class. Persistence in the SF1-restricted comparison reduces concern that the result was driven solely by inclusion of the predominantly TPIT/PIT1-enriched k4 subgroup, but it does not establish common radiomic biology across k3 and k5.

In contrast, k3-focused analyses did not identify statistically significant feature-level or classifier-level separability (Table 2). This is notable because k3 showed prognostic relevance in the reference methylation study, particularly within SF1-lineage tumours [7]. Accordingly, the absence of a stable k3-versus-other radiomic signature suggests that, in this cohort, the relevant molecular differences were not captured by conventional contrast-enhanced T1-weighted MRI. This negative result should also be interpreted in light of the sensitivity of radiomic features to feature-extraction and imaging-protocol choices [13, 14, 25]. Thus, the clinically relevant imaging signal may be stronger at the broader high-risk versus low-risk level than at the level of individual methylation subgroups. This distinction is important because the present findings do not contradict the biological relevance of k3 but suggest that its molecular phenotype may not translate into a sufficiently distinct conventional MRI radiomic pattern in this cohort.

The models targeted outcome-associated methylation strata rather than individual postoperative outcomes. Direct regrowth modelling would have been underpowered because relatively few events occurred in this high-dimensional cohort, whereas methylation subgroup assignment was available for every included tumour and yielded a nearly even binary grouping. Because the k1/k2 versus k3–k5 risk grouping was established through postsurgical outcome associations in the same source cohort, the present analysis is clinically outcome-anchored. Nevertheless, individual regrowth status, time to regrowth, and progression-free survival were not model endpoints. The result should therefore be interpreted as a radiomic correlate of an outcome-associated molecular classification, not as evidence that MRI independently predicts postoperative regrowth. Clinical utility will require larger, independent outcome-linked cohorts demonstrating added value beyond existing clinical and radiological factors.

The inherited outcome association of the molecular labels does not establish prognostic validity of the radiomic classifier. Demonstrating that the classifier predicts postoperative regrowth would require direct analysis of individual outcomes with follow-up time, appropriate censoring, and external validation.

There is a growing body of literature applying radiomics to pituitary tumours. Prior studies have explored MRI radiomics for pituitary tumour recurrence, residual tumour regrowth, consistency, radiosurgical response, proliferative status, visual recovery, and other clinically relevant endpoints [12, 18–20, 26–29]. Pituitary radiomics is promising but still developing, with substantial heterogeneity in study aims, imaging sequences, segmentation strategies, feature-selection methods, and validation approaches [11]. The present study extends this literature by using outcome-associated methylation-defined subgroup and risk-stratum labels as the biological target, rather than individual clinical recurrence or postoperative regrowth endpoints.

Several methodological features strengthen the analysis. First, the radiomic labels were derived from a previously published methylation classification rather than arbitrary imaging-defined groups [7]. Second, the segmentation fine-tuning cases were excluded from the radiomic cohort, reducing the risk of mask-quality leakage. Third, all downstream radiomic analyses used automated masks, reflecting the intended deployment setting. Fourth, statistical inference combined feature-level testing, multiple-testing correction, internally cross-validated classifier performance, and empirical permutation testing. Finally, the analysis hierarchy was biologically motivated: the primary analysis tested the broader low-risk versus high-risk structure established in the reference methylation study, while the SF1-restricted and k3-focused analyses addressed lineage-specific and subgroup-specific questions [7].

This study has important limitations. The cohort was single-centre and modest in size, particularly for individual methylation subgroups, and no independent external radiomic validation cohort was available. Although the included and excluded patients had similar methylation-subgroup and risk-stratum distributions, eligibility depended on availability of a compatible preoperative whole-brain MRI; selection related to tertiary-care imaging pathways or unmeasured clinical factors therefore cannot be excluded. The effective events-per-variable was low relative to the candidate feature space, so absolute performance estimates should be interpreted cautiously despite leakage-controlled cross-validation, permutation testing, and bootstrap optimism correction. Because the low-risk/high-risk strata were defined through postsurgical outcome associations in the same source cohort from which the 74-patient radiomic subset was selected, the present analysis is not an independent validation of prognostic utility. The models classified these inherited outcome-associated labels rather than postoperative regrowth or progression-free survival directly; therefore, the clinical value of this imaging signal remains to be established in independent outcome-linked cohorts. Histopathological correlates such as Ki-67, vascularity, cystic change, or haemorrhage were not available to biologically ground the radiomic features. Although re-extraction with a pinned IBSI-verified PyRadiomics release reproduced all features with ICC ≥ 0.90 and yielded an essentially unchanged optimism-corrected primary AUC, the radiomic workflow remains internally validated only. In addition, the exploratory five-class analysis was conducted using a separate earlier multiclass framework and should not be directly compared with the four binary analyses evaluated under the locked pipeline.

Although all examinations were acquired on Philips systems and 71 of 74 used the dominant protocol, acquisition was not identical: seven examinations were performed at 3 T, native spatial resolution varied, and three examinations used minority protocols, including one 2D acquisition. Isotropic resampling, foreground intensity normalisation and fixed-bin discretisation standardised geometry and intensity scale but cannot eliminate scanner- or sequence-dependent radiomic effects; no statistical batch harmonisation was applied. Excluding the three minority-protocol examinations produced modestly attenuated but still significant performance, which reduces concern that these cases alone drove the primary result. However, only seven 3T examinations were available, making field-strength-specific estimates and ComBat-type harmonisation unreliable. Residual acquisition-related confounding therefore remains possible and should be addressed in larger, prospectively harmonised multicentre cohorts.

The exclusive use of contrast-enhanced T1-weighted MRI was therefore intentional and reflected the routine preoperative neuronavigation workflow represented by the cohort. This pragmatic design tested the potential value of an image already acquired and available during surgical planning, but it does not imply that T1-weighted imaging is the optimal sequence for molecular characterisation. T2-weighted, diffusion-weighted, perfusion-weighted and other quantitative sequences may provide complementary information regarding tumour composition, cellularity, cystic or haemorrhagic change and tissue heterogeneity. Future studies should determine whether multiparametric MRI provides incremental value over this pragmatic T1-weighted baseline.

Segmentation performance was quantified using held-out predictions in the 20 manually annotated cases, but case-level anatomical accuracy could not be established in the 74-patient radiomic cohort because manual reference masks and systematic visual adjudication were unavailable. Automated technical quality control identified no empty, very small or spacing-invalid masks; however, these checks cannot detect anatomically plausible under-segmentation, over-segmentation or boundary inaccuracies. Such errors may affect shape, first-order and texture features and could consequently influence feature stability and classification performance. Segmentation uncertainty therefore remains an important limitation requiring evaluation in future cohorts with case-level visual review, independent manual reference masks and segmentation-perturbation analyses.

Future studies should optimise imaging and parameter choices before validating these findings in independent, preferably multicentre cohorts with harmonised MRI acquisition, segmentation quality assessment, and linkage to longitudinal clinical outcomes [11, 16, 17, 25]. Multiparametric MRI may be needed to capture subgroup-specific biology such as k3-associated regrowth risk, as pituitary imaging studies increasingly suggest that quantitative features derived from additional MRI sequences may reflect clinically relevant tumour characteristics [11, 26–29]. Integration of radiomic, methylation, transcriptomic, histopathological, and clinicopathological data may ultimately provide a more complete risk model for NFPA management [5–8, 24]. Such multimodal integration would align with emerging prognostic frameworks that combine clinical, radiological, pathological, and molecular information for pituitary tumour risk stratification [5–8].

In conclusion, outcome-associated methylation-defined risk labels in NFPAs were associated, at the group level, with the preoperative MRI radiomic phenotype. The strongest signal was observed for the composite high-risk versus low-risk distinction and persisted within SF1-lineage tumours after exclusion of the predominantly TPIT/PIT1-enriched k4 subgroup. Because the high-risk class pooled molecularly distinct k3, k4, and k5 tumours and k3-specific separation was not achieved, the study does not establish a common radiomic signature of aggressiveness across the high-risk subgroups. As the risk grouping inherited its postsurgical outcome association from the same source cohort, the study was clinically outcome-anchored; however, the radiomic model classified the molecular grouping and did not directly model individual postoperative outcomes. These findings provide proof-of-concept evidence that standard preoperative MRI may contain non-invasive radiomic correlates of outcome-associated methylation phenotypes. Independent, outcome-linked validation is required before this approach can support preoperative prognostic assessment.

## Supplementary information

Below is the link to the electronic supplementary material.

Supplementary Material 1 (DOCX 907 KB)

## Data Availability

No datasets were generated or analysed during the current study.
