# Supplementary material for: Association between preoperative MRI radiomic features and methylation-defined phenotypes in non-functioning pituitary adenomas
Source: Pituitary. 2026 Aug 31;29(5):152. doi: 10.1007/s11102-026-01755-w (PMC13529862; doi:10.1007/s11102-026-01755-w)
Supplement: Supplementary file 1 [file 11102_2026_1755_MOESM1_ESM.docx]

**Supplementary Material**

**Supplementary Methods**

**Automated tumour segmentation**

Automated tumour segmentation was performed using a two-dimensional slice-based U-Net implemented in TensorFlow 2.21.0 with tf_keras 2.21.0. The model was initialised from weights pretrained on contrast-enhanced pituitary MRI data from the University Hospital Zurich Neurosurgical Toolkit group^22^. Input images consisted of axial two-dimensional slices resized to 240 × 240 pixels and min–max normalised to the range [0, 1].

Fine-tuning was performed in two phases. In Phase 1, the 20 manually annotated NFPA cases were divided into five folds. In each fold, 16 cases were used for training and four held-out cases were used for validation, ensuring that each annotated case was evaluated exactly once by a model not trained on that case. Training-fold augmentation included horizontal flipping and brightness jitter applied to image-mask pairs where appropriate. Validation data were not augmented. Segmentation performance was quantified from held-out Phase 1 predictions using volumetric three-dimensional Dice similarity coefficient and percentage volume error.

In Phase 2, five deployment models were fine-tuned on all 20 manually annotated cases. These models were not used to estimate segmentation performance. For each radiomic cohort case, the five deployment models generated probability maps that were averaged to form an ensemble probability map. Binary tumour masks were generated by thresholding the mean probability map at 0.5. All radiomic analyses used these automatically generated Phase 2 ensemble masks. Manual masks were not used for radiomic feature extraction.

**Radiomic feature extraction**

Radiomic features were extracted using PyRadiomics (development build 0.1.dev1+g8ed579383, commit 8ed579383, Python 3.12-compatible), and re-extracted with the pinned released version 3.1.0 as an IBSI sensitivity analysis; the two extractions agreed to ICC ≥ 0.90 for all features

Features were extracted from the original image and from Laplacian of Gaussian-filtered images with σ = 1.0, 2.0, and 3.0 mm. Wavelet-filtered images were not included to limit dimensionality relative to cohort size. Extracted feature classes included first-order statistics, shape, grey-level co-occurrence matrix, grey-level run-length matrix, grey-level size-zone matrix, grey-level dependence matrix, and neighbouring grey-tone difference matrix features. A total of 386 radiomic features were extracted per case.

**Feature reduction and statistical testing**

For cohort-level feature testing, radiomic redundancy was reduced using Spearman correlation clustering with an absolute correlation threshold of |r| ≥ 0.90. One deterministic representative feature was retained from each cluster. This yielded 164 retained features for the primary binary, five-class, SF1-restricted and k3-versus-rest feature-level analyses and 165 retained features for the k3-versus-low-risk analysis. These globally retained sets were used only for cohort-level univariable statistical testing. For predictive binary modelling, standardisation and Spearman correlation clustering were refitted independently within each outer training fold and were therefore allowed to vary across folds.

Univariable group differences in retained radiomic features were assessed using Kruskal–Wallis testing. Effect sizes were estimated as η², with uncertainty quantified using 1,000 bootstrap iterations to derive 95% confidence intervals. Multiple testing was controlled using Benjamini–Hochberg false discovery rate correction, with q < 0.05 considered statistically significant. Bonferroni correction was also reported as a stricter secondary criterion. Where relevant, post-hoc comparisons used Dunn tests with Holm correction, and pairwise effect sizes were characterised using Cliff’s δ.

**Machine-learning classification**

Four binary classification analyses were evaluated under the locked framework: primary high-risk versus low-risk classification, SF1-restricted high-risk versus low-risk classification, k3 versus all other subgroups, and k3 versus the low-risk subgroups. Repeated stratified five-fold cross-validation with three repeats was used, corresponding to 15 outer-fold evaluations. Balanced accuracy was the primary performance metric, and AUC was also reported.

Within each outer training fold, features were standardised using parameters estimated from the training data and reduced using label-independent Spearman correlation clustering. The same fitted transformations were then applied to the corresponding test fold. The primary classifier was L1-penalised logistic regression. The regularisation parameter C was selected by three-fold inner cross-validation from the candidate values 0.01, 0.03, 0.1, 0.3 and 1.0, using balanced accuracy as the selection criterion and class weighting to address imbalance. The L1 penalty provided embedded feature selection.

A secondary six-family classifier-selection procedure evaluated L1-penalised logistic regression, random forest, calibrated linear support vector machine, shrinkage linear discriminant analysis, CatBoost and XGBoost. Within each outer training fold, the classifier family was selected according to mean balanced accuracy in three-fold inner cross-validation. No outer test-fold performance was used for classifier selection.

Statistical significance for the binary analyses was assessed using whole-pipeline permutation testing with 1,000 label shuffles. For every shuffle, all preprocessing, model tuning, model fitting and repeated cross-validation steps were rerun. The empirical p value was calculated as the proportion of permuted mean balanced-accuracy values greater than or equal to the observed value, with +1 smoothing. For the primary and SF1-restricted analyses, the complete six-family classifier-selection procedure was also repeated within each permutation.

MRI-acquisition sensitivity analysis. To assess whether minority acquisition protocols influenced the primary result, the complete prespecified L1-penalised logistic-regression pipeline was rerun after excluding the three minority-protocol examinations, leaving 71 patients. The same repeated stratified five-fold cross-validation with three repeats, in-fold preprocessing and regularisation tuning, bootstrap confidence intervals, .632+ optimism correction, and 1,000-shuffle whole-pipeline permutation test were used. Protocol group was tested for association with methylation-risk stratum using a two-sided Fisher exact test.

A separate exploratory five-class k1–k5 analysis was conducted using CatBoost and 500-shuffle permutation testing. This multiclass analysis was not rerun under the locked binary-classification framework and is reported separately.

**Software environment**

Analyses were performed in Python 3.12.3 on Ubuntu Linux. Deep learning used TensorFlow 2.21.0 with tf_keras 2.21.0. Radiomic feature extraction used PyRadiomics 0.1.dev1+g8ed579383, SimpleITK, and nibabel. Statistical analyses and machine-learning models used scikit-learn, SciPy, statsmodels, CatBoost, and XGBoost. Additional packages used for data handling and visualisation included pandas, NumPy, matplotlib, and seaborn. Full package versions are listed in Supplementary Table S2.

**Supplementary Table S1 - Detailed MRI Acquisition Protocol**

Purpose: To document MRI acquisition parameters across the radiomic cohort and allow readers to assess limited residual acquisition variability relevant to radiomic feature extraction.

| **Protocol variant** | **Scanner vendor/model** | **Field strength** | **Sequence type** | **Contrast** | **TR (ms)** | **TE (ms)** | **Flip angle (°)** | **Slice thickness (mm)** | **In-plane resolution (mm × mm)** | **Matrix** | **Number of cases** |
| --- | --- | --- | --- | --- | --- | --- | --- | --- | --- | --- | --- |
| Dominant protocol | Philips | 1.5 T (n=65) / 3 T (n=6) | 3D T1-weighted fast-field echo | Gadolinium-enhanced | 20–25 | 3.8–4.6 | 30 | 1.0–2.0 (native; all resampled to 1.0 mm isotropic before extraction) | 0.868–0.977 × 0.868–0.977 (native; resampled to 1.0 × 1.0 mm) | 256×256–336×336 | 71 |
| Minority protocol | Philips | 1.5 T (n=2) / 3 T (n=1) | T1W_SPIR hals SENSE; t1_se_tra_gd (2D SE); T1W gd CLEAR | Gadolinium-enhanced | 524–617 | 7.8–18 | 62–90 | 3.3–6.0 (native; resampled to 1.0 mm isotropic before extraction) | 0.719–0.898 × 0.719–0.898 (native; resampled to 1.0 × 1.0 mm) | 288×288–384×288 | 3 |

Notes: Most examinations were acquired at 1.5 T (n=67); seven cases were acquired at 3 T (dominant protocol n=6, minority protocol n=1). Native pre-resampling spacings were extracted from PyRadiomics extraction log warnings. Dominant protocol (n = 71): 3D T1-weighted FFE variants (T1W-STEALTH CLEAR and related sequences); native in-plane resolution 0.868–0.977 mm, native slice thickness 1.0–2.0 mm. Minority protocols (n = 3): three cases with native slice thickness ≥ 3.3 mm (T1W_SPIR hals SENSE: 0.729 × 0.729 × 3.3 mm; t1_se_tra_gd [2D SE]: 0.719 × 0.719 × 6.0 mm; T1W gd CLEAR: 0.898 × 0.898 × 6.0 mm). All images and masks were resampled to a common 1.0 × 1.0 × 1.0 mm isotropic grid before radiomic feature extraction. TR, TE, flip angle, and matrix size confirmed from direct DICOM header parsing. Flip angle stored as 62° for T1W_SE CLEAR (Philips SE encoding); nominal SE excitation flip angle is 90°. Image intensities were z-score normalised within the non-zero foreground, and radiomic discretisation used a fixed bin count of 32. No scanner-, field-strength- or sequence-specific statistical batch harmonisation, such as ComBat, was applied.

**Supplementary Table S2 - Pipeline Parameter Details**

**S2A. Segmentation Training and Deployment Parameters**

| **Parameter** | **Phase 1: internal held-out evaluation** | **Phase 2: deployment ensemble** |
| --- | --- | --- |
| Segmentation model | 2D slice-based U-Net | 2D slice-based U-Net |
| Implementation | TensorFlow 2.21.0 with tf_keras 2.21.0 | TensorFlow 2.21.0 with tf_keras 2.21.0 |
| Initialisation | Weights pretrained on contrast-enhanced pituitary MRI data from the University Hospital Zurich Neurosurgical Toolkit group | Same pretrained initialisation |
| Input orientation | Axial 2D slices | Axial 2D slices |
| Input size | 240 × 240 pixels | 240 × 240 pixels |
| Intensity preprocessing | Min-max normalisation to [0, 1] | Min-max normalisation to [0, 1] |
| Number of manually annotated cases | 20 | 20 |
| Cross-validation design | Five folds; 16 training cases and four held-out validation cases per fold | Not used for performance estimation |
| Held-out evaluation | Each annotated case evaluated exactly once by a model not trained on that case | Not applicable |
| Training data augmentation | Horizontal flipping and brightness jitter applied to image-mask pairs where appropriate | Horizontal flipping and brightness jitter applied to image–mask pairs (same as Phase 1; 4× augmentation applied, 894 → 4470 slices per deployment model) |
| Augmentation applied to validation data | No | Not applicable |
| Loss function | Combined Dice loss and binary cross-entropy | Combined Dice loss and binary cross-entropy |
| Optimiser | Adam | Adam |
| Learning-rate strategy | Learning-rate reduction on plateau (ReduceLROnPlateau; reduction factor 0.5; inferred initial LR ≈ 1 × 10⁻⁵ from log: first reduction to 5 × 10⁻⁶ at epoch 8 [fold 1]) | Same strategy; Phase 2 ran to epoch 50 (no validation set — LR schedule applied to training loss; model weights restored from best training epoch) |
| Early stopping | Yes; restoration of best validation weights (Phase 1: fold 1 stopped at epoch 13 [best epoch 5]; fold 2 stopped at epoch 23 [best epoch 15]) | Yes; restoration of best training epoch weights (best epoch 50 for all 5 deployment models, indicating training loss continued to improve through all 50 epochs) |
| Deployment prediction | Not used for radiomic cohort masks | Average probability map from five models, thresholded at 0.5 |
| Threshold for binary mask | 0.5 | 0.5 |
| Performance reporting | Volumetric 3D Dice similarity coefficient and percentage volume error from held-out Phase 1 predictions | Not used for performance reporting |

**S2B. Phase 1 Per-Fold Case and Slice Counts**

| **Fold** | **Training cases** | **Held-out validation cases** | **Training slices** | **Validation slices** |
| --- | --- | --- | --- | --- |
| 1 | 16 | 4 | 730 (3,650 after 4× augmentation) | 1,024 |
| 2 | 16 | 4 | 704 (3,520 after 4× augmentation) | 1,024 |
| 3 | 16 | 4 | Not retained | Not recorded |
| 4 | 16 | 4 | Not retained | Not recorded |
| 5 | 16 | 4 | Not retained | Not recorded |

Notes: Training-fold slice counts are reported after preprocessing and exclusion of non-informative slices. Per-fold slice counts were available for folds 1 and 2; corresponding slice-count logs for folds 3–5 were not retained. However, Phase 1 held-out five-fold evaluation was completed for all 20 manually annotated cases, as confirmed by the availability of held-out predictions and case-level DSC values for every annotated case. Phase 2 deployment models were trained separately on all 20 annotated cases, using 894 original slices augmented to 4,470 slices per deployment model. All five deployment models completed training and were used to generate ensemble probability maps for the radiomic cohort.

**S2C. Radiomic Feature Extraction Configuration**

| **Parameter** | **Setting** |
| --- | --- |
| Software | PyRadiomics 0.1.dev1+g8ed579383 |
| Commit | 8ed579383; AIM-Harvard/pyradiomics |
| Python compatibility | Python 3.12-compatible development build of the v3.x series |
| Input image | Preoperative gadolinium-enhanced T1-weighted MRI |
| Input mask | Automatically generated Phase 2 ensemble tumour mask |
| Manual masks used for radiomic extraction | No |
| Resampling before extraction | 1-mm isotropic image and mask grid |
| Additional PyRadiomics resampling | None |
| Intensity normalisation | Z-score normalisation within the non-zero foreground region |
| Intensity discretisation | Fixed bin count |
| Bin count | 32 |
| Image types | Original image; Laplacian of Gaussian-filtered images |
| LoG sigma values | σ = 1.0, 2.0, and 3.0 mm |
| Wavelet features | Not included |
| Feature classes | First-order statistics; shape; GLCM; GLRLM; GLSZM; GLDM; NGTDM |
| Total extracted features per case | 386 |
| Redundancy reduction | Spearman correlation clustering |
| Correlation threshold | \|r\| ≥ 0.90 |
| Retained features | Cohort-level univariable feature sets: 164 representatives for the primary binary, five-class, SF1-restricted and k3-versus-rest analyses; 165 for the k3-versus-low-risk analysis. For predictive binary modelling, correlation reduction was refitted within each training fold. |

**Supplementary Table S3 – Full Classification Performance for the Four Binary Analyses**

Purpose: To report classifier-specific performance for the four binary radiomic classification tasks under the leakage-controlled pipeline and the classifier-selection frequency across cross-validation folds for the secondary six-family procedure.

**S3A. Classifier-by-Classifier Performance**

| **Analysis** | **Classifier** | **Balanced accuracy [95% CI]** | **AUC [95% CI]** |
| --- | --- | --- | --- |
| Primary binary: high-risk vs low-risk | L1-logistic | 0.750 [0.65, 0.85] | 0.785 [0.66, 0.89] |
| Primary binary: high-risk vs low-risk | Random Forest | 0.736 [0.63, 0.84] | 0.688 [0.56, 0.82] |
| Primary binary: high-risk vs low-risk | Linear SVM | 0.553 [0.47, 0.64] | 0.594 [0.46, 0.73] |
| Primary binary: high-risk vs low-risk | LDA | 0.696 [0.59, 0.80] | 0.656 [0.53, 0.79] |
| Primary binary: high-risk vs low-risk | CatBoost | 0.711 [0.61, 0.81] | 0.624 [0.49, 0.76] |
| Primary binary: high-risk vs low-risk | XGBoost | 0.684 [0.58, 0.79] | 0.638 [0.50, 0.78] |
| SF1-restricted: high-risk vs low-risk | L1-logistic | 0.735 [0.62, 0.84] | 0.730 [0.60, 0.85] |
| SF1-restricted: high-risk vs low-risk | Random Forest | 0.688 [0.58, 0.80] | 0.679 [0.53, 0.82] |
| SF1-restricted: high-risk vs low-risk | Linear SVM | 0.455 [0.36, 0.55] | 0.535 [0.38, 0.68] |
| SF1-restricted: high-risk vs low-risk | LDA | 0.576 [0.45, 0.70] | 0.594 [0.45, 0.73] |
| SF1-restricted: high-risk vs low-risk | CatBoost | 0.667 [0.55, 0.78] | 0.652 [0.50, 0.79] |
| SF1-restricted: high-risk vs low-risk | XGBoost | 0.640 [0.52, 0.75] | 0.661 [0.51, 0.80] |
| k3 vs low-risk | L1-logistic | 0.512 [0.36, 0.66] | 0.601 [0.42, 0.76] |
| k3 vs low-risk | Random Forest | 0.606 [0.49, 0.73] | 0.660 [0.49, 0.82] |
| k3 vs low-risk | Linear SVM | 0.471 [0.42, 0.50] | 0.494 [0.32, 0.67] |
| k3 vs low-risk | LDA | 0.530 [0.40, 0.68] | 0.513 [0.34, 0.70] |
| k3 vs low-risk | CatBoost | 0.630 [0.49, 0.76] | 0.610 [0.44, 0.78] |
| k3 vs low-risk | XGBoost | 0.645 [0.51, 0.78] | 0.580 [0.40, 0.76] |
| k3 vs all others | L1-logistic | 0.323 [0.22, 0.44] | 0.286 [0.16, 0.43] |
| k3 vs all others | Random Forest | 0.491 [0.47, 0.50] | 0.594 [0.43, 0.75] |
| k3 vs all others | Linear SVM | 0.500 [0.50, 0.50] | 0.452 [0.28, 0.63] |
| k3 vs all others | LDA | 0.543 [0.43, 0.66] | 0.562 [0.41, 0.72] |
| k3 vs all others | CatBoost | 0.492 [0.44, 0.56] | 0.554 [0.40, 0.70] |
| k3 vs all others | XGBoost | 0.550 [0.46, 0.65] | 0.580 [0.42, 0.74] |

Notes: Each candidate classifier was run individually under the locked repeated stratified cross-validation, with standardisation, correlation clustering and feature selection performed within training folds; balanced accuracy and AUC are shown with bootstrap 95% confidence intervals. These are out-of-fold candidate-pool estimates and were not used for model selection: the prespecified primary model is the inner-CV-tuned L1-penalised logistic regression reported in Table 2 (balanced accuracy 0.69). The higher fixed-penalty L1 value shown here (0.75) is not promoted to primary, as that would constitute post-hoc selection on out-of-fold performance. The five-class analysis is multiclass and was not re-run under this single-model pipeline; its values remain as originally reported. Bracketed values indicate 95% confidence intervals across repeated cross-validation estimates.

**S3B. Classifier-Selection Frequency Across Cross-Validation Folds (Best-of-Six Procedure)**

| **Analysis** | **Inner-CV selected classifier** | **Folds selected** | **Selection frequency** |
| --- | --- | --- | --- |
| Primary binary: high-risk vs low-risk | Random Forest | 4 / 15 | 27% |
| Primary binary: high-risk vs low-risk | LDA | 3 / 15 | 20% |
| Primary binary: high-risk vs low-risk | XGBoost | 3 / 15 | 20% |
| Primary binary: high-risk vs low-risk | L1-logistic | 3 / 15 | 20% |
| Primary binary: high-risk vs low-risk | Linear SVM | 1 / 15 | 7% |
| Primary binary: high-risk vs low-risk | CatBoost | 1 / 15 | 7% |
| SF1-restricted: high-risk vs low-risk | Random Forest | 5 / 15 | 33% |
| SF1-restricted: high-risk vs low-risk | XGBoost | 5 / 15 | 33% |
| SF1-restricted: high-risk vs low-risk | LDA | 3 / 15 | 20% |
| SF1-restricted: high-risk vs low-risk | L1-logistic | 1 / 15 | 7% |
| SF1-restricted: high-risk vs low-risk | Linear SVM | 1 / 15 | 7% |
| k3 vs low-risk | CatBoost | 4 / 15 | 27% |
| k3 vs low-risk | Random Forest | 4 / 15 | 27% |
| k3 vs low-risk | LDA | 3 / 15 | 20% |
| k3 vs low-risk | L1-logistic | 2 / 15 | 13% |
| k3 vs low-risk | Linear SVM | 1 / 15 | 7% |
| k3 vs low-risk | XGBoost | 1 / 15 | 7% |
| k3 vs all others | L1-logistic | 5 / 15 | 33% |
| k3 vs all others | LDA | 5 / 15 | 33% |
| k3 vs all others | XGBoost | 2 / 15 | 13% |
| k3 vs all others | CatBoost | 2 / 15 | 13% |
| k3 vs all others | Random Forest | 1 / 15 | 7% |

Notes: Nested cross-validation was performed as a supplementary internal assessment of model-selection stability. Because the cohort was small and each outer test fold contained approximately 15 cases, fold-level estimates should not be interpreted in isolation or as expected external validation performance.

**Supplementary Table S4 - Case-Level Segmentation Performance**

Purpose: To report case-level segmentation performance for all 20 manually annotated cases evaluated during Phase 1 held-out cross-validation.

| **Case ID** | **Volumetric DSC** | **Predicted tumour volume (mm³)** | **Ground-truth tumour volume (mm³)** | **Volume error (%)** | **Absolute volume error (%)** | **Comment** |
| --- | --- | --- | --- | --- | --- | --- |
| Case 1 | 0.7395 | 9,326 | 13,734 | -32.1 | 32.1 | — |
| Case 2 | 0.8922 | 3,521 | 3,054 | +15.3 | 15.3 | — |
| Case 3 | 0.8903 | 2,489 | 2,653 | -6.2 | 6.2 | — |
| Case 4 | 0.9074 | 17,101 | 15,730 | +8.7 | 8.7 | — |
| Case 5 | 0.6824 | 2,527 | 1,661 | +52.1 | 52.1 | — |
| Case 6 | 0.8081 | 2,718 | 2,123 | +28.0 | 28.0 | — |
| Case 7 | 0.494 | 7,819 | 2,873 | +172.2 | 172.2 | Over-segmentation failure |
| Case 8 | 0.8733 | 8,704 | 7,187 | +21.1 | 21.1 | — |
| Case 9 | 0.8311 | 7,063 | 7,443 | -5.1 | 5.1 | — |
| Case 10 | 0.8195 | 4,940 | 5,142 | -3.9 | 3.9 | — |
| Case 11 | 0.9174 | 11,710 | 11,118 | +5.3 | 5.3 | — |
| Case 12 | 0.405 | 132 | 500 | −73.6 | 73.6 | Under-segmentation failure |
| Case 13 | 0.9061 | 7,086 | 7,019 | +1.0 | 1.0 | — |
| Case 14 | 0.8715 | 4,287 | 3,603 | +19.0 | 19.0 | — |
| Case 15 | 0.8628 | 9,148 | 7,409 | +23.5 | 23.5 | — |
| Case 16 | 0.7772 | 3,405 | 2,583 | +31.8 | 31.8 | — |
| Case 17 | 0.8389 | 8,073 | 7,959 | +1.4 | 1.4 | — |
| Case 18 | 0.8756 | 7,935 | 6,989 | +13.5 | 13.5 | — |
| Case 19 | 0.8855 | 6,297 | 6,451 | -2.4 | 2.4 | — |
| Case 20 | 0.8815 | 2,437 | 2,341 | +4.1 | 4.1 | — |
| Median | 0.867 | — | — | — | 14.4 | — |
| Mean | 0.808 | — | — | — | 26.0 | — |
| SD | 0.137 | — | — | — | — | — |
| IQR | 0.800-0.887 | — | — | — | — | — |

Notes: DSC, Dice similarity coefficient. Volume error was calculated as: predicted tumour volume minus ground-truth tumour volume, divided by ground-truth tumour volume, multiplied by 100. Each case was segmented by a model trained without that case. Training-log Dice values were not used for performance reporting.

**Supplementary Table S5 — Feature-Level Statistical Results for FDR-Significant Features**

Purpose: To report the full Kruskal-Wallis H-test statistics, effect sizes (η²), confidence intervals, corrected p-values, and pairwise Cliff's δ effect sizes for all FDR-significant features across the three analyses that yielded significant features.

| **Feature** | **Image type** | **Feature class** | **H-statistic** | **p-value** | **η²** | **95% CI** | **FDR q** | **Bonferroni-adjusted p** | **FDR sig** | **Bonferroni sig** | **Cliff's δ (high vs low)** |
| --- | --- | --- | --- | --- | --- | --- | --- | --- | --- | --- | --- |
| **Primary binary — high-risk vs low-risk (n = 74; 7 FDR-significant, 4 Bonferroni-significant)** |  |  |  |  |  |  |  |  |  |  |  |
| log-sigma-3-0-mm-3D_firstorder_Median | LoG σ = 3.0 mm | First-order | 21.55 | 0.0000035 | 0.285 | [0.115, 0.504] | 0.000565 | 0.000565 | Yes | Yes | 0.629 |
| log-sigma-2-0-mm-3D_firstorder_Median | LoG σ = 2.0 mm | First-order | 19.68 | 0.0000091 | 0.259 | [0.097, 0.460] | 0.000751 | 0.001501 | Yes | Yes | 0.601 |
| log-sigma-3-0-mm-3D_firstorder_90Percentile | LoG σ = 3.0 mm | First-order | 16.37 | 0.0000521 | 0.213 | [0.065, 0.415] | 0.002851 | 0.008553 | Yes | Yes | 0.549 |
| log-sigma-1-0-mm-3D_firstorder_10Percentile | LoG σ = 1.0 mm | First-order | 15.42 | 0.0000862 | 0.200 | [0.055, 0.403] | 0.003535 | 0.014139 | Yes | Yes | 0.532 |
| log-sigma-3-0-mm-3D_firstorder_10Percentile | LoG σ = 3.0 mm | First-order | 12.89 | 0.000330 | 0.165 | [0.029, 0.352] | 0.010838 | 0.054190 | Yes | No | 0.487 |
| log-sigma-3-0-mm-3D_glcm_Imc1 | LoG σ = 3.0 mm | GLCM | 10.52 | 0.001182 | 0.132 | [0.013, 0.310] | 0.032317 | 0.193901 | Yes | No | −0.440 |
| log-sigma-3-0-mm-3D_firstorder_Energy | LoG σ = 3.0 mm | First-order | 10.17 | 0.001428 | 0.127 | [0.013, 0.320] | 0.033462 | 0.234235 | Yes | No | −0.432 |
| **Five-class k1–k5 (n = 74; 3 FDR-significant, 3 Bonferroni-significant)** |  |  |  |  |  |  |  |  |  |  |  |
| log-sigma-3-0-mm-3D_firstorder_Median | LoG σ = 3.0 mm | First-order | 26.30 | 0.0000282 | 0.323 | [0.159, 0.559] | 0.003255 | 0.004522 | Yes | Yes | max \|δ\| = 0.833 (k2 vs k4) |
| log-sigma-3-0-mm-3D_firstorder_90Percentile | LoG σ = 3.0 mm | First-order | 24.95 | 0.0000511 | 0.304 | [0.172, 0.512] | 0.003255 | 0.008427 | Yes | Yes | max \|δ\| = 0.856 (k2 vs k4) |
| log-sigma-2-0-mm-3D_firstorder_Median | LoG σ = 2.0 mm | First-order | 24.64 | 0.0000604 | 0.299 | [0.129, 0.551] | 0.003255 | 0.009764 | Yes | Yes | max \|δ\| = 0.780 (k2 vs k4) |
| **SF1-restricted binary — low-risk vs high-risk, excluding k4 (n = 62; 4 FDR-significant, 3 Bonferroni-significant)** |  |  |  |  |  |  |  |  |  |  |  |
| log-sigma-3-0-mm-3D_firstorder_Median | LoG σ = 3.0 mm | First-order | 15.13 | 0.000100 | 0.236 | [0.068, 0.450] | 0.012866 | 0.016449 | Yes | Yes | 0.578 |
| log-sigma-1-0-mm-3D_firstorder_10Percentile | LoG σ = 1.0 mm | First-order | 14.16 | 0.000168 | 0.219 | [0.054, 0.445] | 0.012866 | 0.027582 | Yes | Yes | 0.559 |
| log-sigma-2-0-mm-3D_firstorder_Median | LoG σ = 2.0 mm | First-order | 13.53 | 0.000235 | 0.209 | [0.051, 0.460] | 0.012866 | 0.038598 | Yes | Yes | 0.546 |
| log-sigma-3-0-mm-3D_firstorder_10Percentile | LoG σ = 3.0 mm | First-order | 10.68 | 0.001085 | 0.161 | [0.023, 0.376] | 0.044488 | 0.177951 | Yes | No | 0.485 |

Notes: η², eta-squared effect size (Kruskal-Wallis); 95% CI from 1,000 bootstrap iterations. FDR correction was performed using the Benjamini-Hochberg procedure, with q < 0.05 considered statistically significant. Bonferroni significance was defined as Bonferroni-adjusted p < 0.05. Cliff's δ: pairwise effect size (high-risk vs low-risk for binary analyses; maximum absolute pairwise value across all subgroup pairs for the five-class analysis, with the specific comparison shown in parentheses). Positive Cliff's δ indicates higher values in the high-risk group.

**Supplementary Table S6 — Complete Kruskal-Wallis Statistical Results: All 164 Retained Features (Primary Binary Analysis)**

Purpose: To report the full Kruskal-Wallis H-test results for all 164 features retained after Spearman correlation clustering in the primary binary high-risk versus low-risk analysis. Features are sorted by η² in descending order.

| **Feature** | **H-statistic** | **p-value** | **η²** | **95% CI (lo–hi)** | **FDR q** | **Bonferroni-adjusted p** | **FDR sig** | **Bonferroni sig** |
| --- | --- | --- | --- | --- | --- | --- | --- | --- |
| log-sigma-3-0-mm-3D_firstorder_Median | 21.551 | 3.445324e-06 | 0.285 | [0.115, 0.504] | 0.000565 | 0.000565 | Yes | Yes |
| log-sigma-2-0-mm-3D_firstorder_Median | 19.680 | 9.154894e-06 | 0.259 | [0.097, 0.460] | 0.000751 | 0.001501 | Yes | Yes |
| log-sigma-3-0-mm-3D_firstorder_90Percentile | 16.368 | 5.215519e-05 | 0.213 | [0.065, 0.415] | 0.002851 | 0.008553 | Yes | Yes |
| log-sigma-1-0-mm-3D_firstorder_10Percentile | 15.417 | 8.621192e-05 | 0.200 | [0.055, 0.403] | 0.003535 | 0.014139 | Yes | Yes |
| log-sigma-3-0-mm-3D_firstorder_10Percentile | 12.889 | 3.304255e-04 | 0.165 | [0.029, 0.352] | 0.010838 | 0.054190 | Yes | No |
| log-sigma-3-0-mm-3D_glcm_Imc1 | 10.518 | 0.001182 | 0.132 | [0.013, 0.310] | 0.032317 | 0.193901 | Yes | No |
| log-sigma-3-0-mm-3D_firstorder_Energy | 10.169 | 0.001428 | 0.127 | [0.013, 0.320] | 0.033462 | 0.234235 | Yes | No |
| log-sigma-3-0-mm-3D_glszm_SmallAreaHighGrayLevelEmphasis | 7.710 | 0.005491 | 0.093 | [-0.004, 0.281] | 0.103452 | 0.900560 | No | No |
| log-sigma-1-0-mm-3D_firstorder_InterquartileRange | 7.650 | 0.005677 | 0.092 | [-0.001, 0.254] | 0.103452 | 0.931066 | No | No |
| original_firstorder_10Percentile | 6.608 | 0.010151 | 0.078 | [-0.006, 0.236] | 0.151350 | 1.000000 | No | No |
| log-sigma-3-0-mm-3D_glszm_HighGrayLevelZoneEmphasis | 6.608 | 0.010151 | 0.078 | [-0.008, 0.234] | 0.151350 | 1.000000 | No | No |
| log-sigma-2-0-mm-3D_glcm_Imc1 | 6.170 | 0.012997 | 0.072 | [-0.010, 0.236] | 0.169023 | 1.000000 | No | No |
| log-sigma-3-0-mm-3D_glcm_Correlation | 6.116 | 0.013398 | 0.071 | [-0.010, 0.253] | 0.169023 | 1.000000 | No | No |
| log-sigma-3-0-mm-3D_firstorder_Minimum | 5.956 | 0.014668 | 0.069 | [-0.009, 0.240] | 0.171828 | 1.000000 | No | No |
| original_firstorder_90Percentile | 5.694 | 0.017022 | 0.065 | [-0.009, 0.217] | 0.186106 | 1.000000 | No | No |
| log-sigma-3-0-mm-3D_ngtdm_Complexity | 5.438 | 0.019701 | 0.062 | [-0.013, 0.229] | 0.201931 | 1.000000 | No | No |
| log-sigma-2-0-mm-3D_ngtdm_Complexity | 4.848 | 0.027676 | 0.053 | [-0.011, 0.199] | 0.246488 | 1.000000 | No | No |
| original_firstorder_Skewness | 4.706 | 0.030060 | 0.051 | [-0.013, 0.209] | 0.246488 | 1.000000 | No | No |
| original_glrlm_LongRunHighGrayLevelEmphasis | 4.706 | 0.030060 | 0.051 | [-0.013, 0.214] | 0.246488 | 1.000000 | No | No |
| log-sigma-1-0-mm-3D_firstorder_Range | 4.706 | 0.030060 | 0.051 | [-0.012, 0.194] | 0.246488 | 1.000000 | No | No |
| log-sigma-3-0-mm-3D_firstorder_Kurtosis | 4.473 | 0.034424 | 0.048 | [-0.013, 0.195] | 0.268834 | 1.000000 | No | No |
| log-sigma-3-0-mm-3D_glrlm_RunEntropy | 4.247 | 0.039318 | 0.045 | [-0.012, 0.192] | 0.293101 | 1.000000 | No | No |
| original_glcm_Autocorrelation | 3.897 | 0.048374 | 0.040 | [-0.013, 0.194] | 0.344928 | 1.000000 | No | No |
| log-sigma-2-0-mm-3D_firstorder_Minimum | 3.521 | 0.060594 | 0.035 | [-0.013, 0.183] | 0.414057 | 1.000000 | No | No |
| log-sigma-3-0-mm-3D_gldm_DependenceEntropy | 3.400 | 0.065196 | 0.033 | [-0.014, 0.177] | 0.427689 | 1.000000 | No | No |
| log-sigma-3-0-mm-3D_glszm_LowGrayLevelZoneEmphasis | 3.242 | 0.071778 | 0.031 | [-0.013, 0.180] | 0.440854 | 1.000000 | No | No |
| log-sigma-3-0-mm-3D_glcm_ClusterTendency | 3.203 | 0.073506 | 0.031 | [-0.013, 0.168] | 0.440854 | 1.000000 | No | No |
| original_shape_Sphericity | 3.164 | 0.075268 | 0.030 | [-0.013, 0.184] | 0.440854 | 1.000000 | No | No |
| original_gldm_LargeDependenceHighGrayLevelEmphasis | 2.974 | 0.084600 | 0.027 | [-0.013, 0.144] | 0.478428 | 1.000000 | No | No |
| original_glcm_ClusterShade | 2.863 | 0.090635 | 0.026 | [-0.014, 0.161] | 0.495471 | 1.000000 | No | No |
| original_glrlm_LongRunLowGrayLevelEmphasis | 2.718 | 0.099215 | 0.024 | [-0.014, 0.168] | 0.524879 | 1.000000 | No | No |
| log-sigma-3-0-mm-3D_glszm_SmallAreaLowGrayLevelEmphasis | 2.372 | 0.123510 | 0.019 | [-0.014, 0.158] | 0.603919 | 1.000000 | No | No |
| original_gldm_LargeDependenceLowGrayLevelEmphasis | 2.372 | 0.123510 | 0.019 | [-0.014, 0.150] | 0.603919 | 1.000000 | No | No |
| log-sigma-2-0-mm-3D_glcm_Correlation | 2.306 | 0.128885 | 0.018 | [-0.014, 0.146] | 0.603919 | 1.000000 | No | No |
| log-sigma-3-0-mm-3D_firstorder_Range | 2.306 | 0.128885 | 0.018 | [-0.014, 0.163] | 0.603919 | 1.000000 | No | No |
| log-sigma-1-0-mm-3D_firstorder_Energy | 2.208 | 0.137286 | 0.017 | [-0.014, 0.142] | 0.625414 | 1.000000 | No | No |
| log-sigma-3-0-mm-3D_gldm_SmallDependenceHighGrayLevelEmphasis | 2.144 | 0.143117 | 0.016 | [-0.014, 0.140] | 0.634358 | 1.000000 | No | No |
| original_firstorder_InterquartileRange | 2.050 | 0.152218 | 0.015 | [-0.014, 0.145] | 0.656941 | 1.000000 | No | No |
| log-sigma-3-0-mm-3D_gldm_GrayLevelVariance | 1.988 | 0.158526 | 0.014 | [-0.014, 0.130] | 0.666620 | 1.000000 | No | No |
| log-sigma-1-0-mm-3D_gldm_LargeDependenceHighGrayLevelEmphasis | 1.897 | 0.168355 | 0.013 | [-0.014, 0.136] | 0.683950 | 1.000000 | No | No |
| log-sigma-1-0-mm-3D_glszm_GrayLevelVariance | 1.838 | 0.175158 | 0.012 | [-0.014, 0.126] | 0.683950 | 1.000000 | No | No |
| log-sigma-1-0-mm-3D_glszm_SizeZoneNonUniformityNormalized | 1.838 | 0.175158 | 0.012 | [-0.014, 0.151] | 0.683950 | 1.000000 | No | No |
| original_firstorder_Range | 1.780 | 0.182164 | 0.011 | [-0.014, 0.119] | 0.694764 | 1.000000 | No | No |
| log-sigma-1-0-mm-3D_firstorder_Skewness | 1.722 | 0.189375 | 0.010 | [-0.014, 0.130] | 0.705854 | 1.000000 | No | No |
| log-sigma-2-0-mm-3D_firstorder_InterquartileRange | 1.611 | 0.204426 | 0.009 | [-0.014, 0.125] | 0.745018 | 1.000000 | No | No |
| log-sigma-2-0-mm-3D_firstorder_Range | 1.398 | 0.237098 | 0.005 | [-0.014, 0.129] | 0.839863 | 1.000000 | No | No |
| log-sigma-2-0-mm-3D_glrlm_RunEntropy | 1.347 | 0.245814 | 0.005 | [-0.014, 0.111] | 0.839863 | 1.000000 | No | No |
| log-sigma-1-0-mm-3D_glszm_SizeZoneNonUniformity | 1.347 | 0.245814 | 0.005 | [-0.014, 0.110] | 0.839863 | 1.000000 | No | No |
| log-sigma-1-0-mm-3D_glrlm_LowGrayLevelRunEmphasis | 1.297 | 0.254751 | 0.004 | [-0.014, 0.104] | 0.852637 | 1.000000 | No | No |
| original_glszm_GrayLevelVariance | 1.153 | 0.282910 | 0.002 | [-0.014, 0.112] | 0.913435 | 1.000000 | No | No |
| original_glszm_HighGrayLevelZoneEmphasis | 1.084 | 0.297752 | 0.001 | [-0.014, 0.112] | 0.913435 | 1.000000 | No | No |
| log-sigma-2-0-mm-3D_glszm_GrayLevelVariance | 1.062 | 0.302813 | 0.001 | [-0.014, 0.103] | 0.913435 | 1.000000 | No | No |
| original_ngtdm_Complexity | 1.040 | 0.307931 | 0.001 | [-0.014, 0.098] | 0.913435 | 1.000000 | No | No |
| original_glrlm_RunEntropy | 1.040 | 0.307931 | 0.001 | [-0.014, 0.110] | 0.913435 | 1.000000 | No | No |
| original_glcm_Contrast | 1.018 | 0.313106 | 0.000 | [-0.014, 0.114] | 0.913435 | 1.000000 | No | No |
| original_shape_SurfaceArea | 0.911 | 0.339834 | -0.001 | [-0.014, 0.104] | 0.913435 | 1.000000 | No | No |
| original_shape_Maximum3DDiameter | 0.890 | 0.345350 | -0.002 | [-0.014, 0.092] | 0.913435 | 1.000000 | No | No |
| log-sigma-3-0-mm-3D_gldm_LargeDependenceHighGrayLevelEmphasis | 0.870 | 0.350923 | -0.002 | [-0.014, 0.104] | 0.913435 | 1.000000 | No | No |
| log-sigma-1-0-mm-3D_gldm_SmallDependenceLowGrayLevelEmphasis | 0.830 | 0.362239 | -0.002 | [-0.014, 0.092] | 0.913435 | 1.000000 | No | No |
| original_shape_Maximum2DDiameterColumn | 0.811 | 0.367956 | -0.003 | [-0.014, 0.092] | 0.913435 | 1.000000 | No | No |
| original_glcm_JointEntropy | 0.772 | 0.379635 | -0.003 | [-0.014, 0.095] | 0.913435 | 1.000000 | No | No |
| log-sigma-2-0-mm-3D_glcm_MaximumProbability | 0.772 | 0.379635 | -0.003 | [-0.014, 0.104] | 0.913435 | 1.000000 | No | No |
| original_gldm_SmallDependenceHighGrayLevelEmphasis | 0.734 | 0.391514 | -0.004 | [-0.014, 0.100] | 0.913435 | 1.000000 | No | No |
| original_firstorder_Energy | 0.734 | 0.391514 | -0.004 | [-0.014, 0.091] | 0.913435 | 1.000000 | No | No |
| log-sigma-1-0-mm-3D_glcm_Autocorrelation | 0.716 | 0.397537 | -0.004 | [-0.014, 0.095] | 0.913435 | 1.000000 | No | No |
| log-sigma-1-0-mm-3D_glcm_MCC | 0.716 | 0.397537 | -0.004 | [-0.014, 0.091] | 0.913435 | 1.000000 | No | No |
| log-sigma-3-0-mm-3D_glcm_ClusterProminence | 0.716 | 0.397537 | -0.004 | [-0.014, 0.098] | 0.913435 | 1.000000 | No | No |
| original_glszm_GrayLevelNonUniformityNormalized | 0.679 | 0.409749 | -0.004 | [-0.014, 0.088] | 0.913435 | 1.000000 | No | No |
| log-sigma-2-0-mm-3D_glcm_Contrast | 0.662 | 0.415938 | -0.005 | [-0.014, 0.092] | 0.913435 | 1.000000 | No | No |
| log-sigma-2-0-mm-3D_glszm_HighGrayLevelZoneEmphasis | 0.662 | 0.415938 | -0.005 | [-0.014, 0.097] | 0.913435 | 1.000000 | No | No |
| original_glcm_DifferenceAverage | 0.644 | 0.422181 | -0.005 | [-0.014, 0.091] | 0.913435 | 1.000000 | No | No |
| log-sigma-2-0-mm-3D_gldm_DependenceEntropy | 0.627 | 0.428480 | -0.005 | [-0.014, 0.080] | 0.913435 | 1.000000 | No | No |
| log-sigma-1-0-mm-3D_glcm_Contrast | 0.627 | 0.428480 | -0.005 | [-0.014, 0.087] | 0.913435 | 1.000000 | No | No |
| log-sigma-1-0-mm-3D_glrlm_LongRunLowGrayLevelEmphasis | 0.627 | 0.428480 | -0.005 | [-0.014, 0.084] | 0.913435 | 1.000000 | No | No |
| original_glcm_Imc2 | 0.610 | 0.434832 | -0.005 | [-0.014, 0.093] | 0.913435 | 1.000000 | No | No |
| log-sigma-2-0-mm-3D_glcm_DifferenceVariance | 0.593 | 0.441239 | -0.006 | [-0.014, 0.076] | 0.913435 | 1.000000 | No | No |
| log-sigma-3-0-mm-3D_glcm_DifferenceAverage | 0.577 | 0.447699 | -0.006 | [-0.014, 0.094] | 0.913435 | 1.000000 | No | No |
| log-sigma-1-0-mm-3D_gldm_LargeDependenceEmphasis | 0.577 | 0.447699 | -0.006 | [-0.014, 0.095] | 0.913435 | 1.000000 | No | No |
| log-sigma-3-0-mm-3D_glszm_SmallAreaEmphasis | 0.560 | 0.454213 | -0.006 | [-0.014, 0.076] | 0.913435 | 1.000000 | No | No |
| original_shape_Flatness | 0.560 | 0.454213 | -0.006 | [-0.014, 0.079] | 0.913435 | 1.000000 | No | No |
| log-sigma-2-0-mm-3D_firstorder_Kurtosis | 0.544 | 0.460780 | -0.006 | [-0.014, 0.076] | 0.913435 | 1.000000 | No | No |
| original_shape_Maximum2DDiameterSlice | 0.528 | 0.467370 | -0.007 | [-0.014, 0.082] | 0.913435 | 1.000000 | No | No |
| log-sigma-1-0-mm-3D_glcm_DifferenceAverage | 0.512 | 0.474071 | -0.007 | [-0.014, 0.078] | 0.913435 | 1.000000 | No | No |
| log-sigma-3-0-mm-3D_glszm_SizeZoneNonUniformity | 0.497 | 0.480795 | -0.007 | [-0.014, 0.078] | 0.913435 | 1.000000 | No | No |
| log-sigma-3-0-mm-3D_glszm_GrayLevelVariance | 0.497 | 0.480795 | -0.007 | [-0.014, 0.097] | 0.913435 | 1.000000 | No | No |
| log-sigma-3-0-mm-3D_glcm_ClusterShade | 0.467 | 0.494398 | -0.007 | [-0.014, 0.084] | 0.913435 | 1.000000 | No | No |
| log-sigma-1-0-mm-3D_firstorder_Maximum | 0.467 | 0.494398 | -0.007 | [-0.014, 0.088] | 0.913435 | 1.000000 | No | No |
| log-sigma-1-0-mm-3D_glszm_HighGrayLevelZoneEmphasis | 0.452 | 0.501275 | -0.008 | [-0.014, 0.080] | 0.913435 | 1.000000 | No | No |
| log-sigma-3-0-mm-3D_firstorder_InterquartileRange | 0.452 | 0.501275 | -0.008 | [-0.014, 0.078] | 0.913435 | 1.000000 | No | No |
| log-sigma-2-0-mm-3D_glcm_JointEntropy | 0.452 | 0.501275 | -0.008 | [-0.014, 0.082] | 0.913435 | 1.000000 | No | No |
| original_ngtdm_Contrast | 0.438 | 0.508203 | -0.008 | [-0.014, 0.074] | 0.915883 | 1.000000 | No | No |
| log-sigma-2-0-mm-3D_gldm_SmallDependenceHighGrayLevelEmphasis | 0.396 | 0.529285 | -0.008 | [-0.014, 0.079] | 0.916366 | 1.000000 | No | No |
| log-sigma-3-0-mm-3D_glcm_Contrast | 0.396 | 0.529285 | -0.008 | [-0.014, 0.075] | 0.916366 | 1.000000 | No | No |
| log-sigma-1-0-mm-3D_glszm_LargeAreaHighGrayLevelEmphasis | 0.396 | 0.529285 | -0.008 | [-0.014, 0.073] | 0.916366 | 1.000000 | No | No |
| log-sigma-1-0-mm-3D_glszm_LowGrayLevelZoneEmphasis | 0.382 | 0.536409 | -0.009 | [-0.014, 0.080] | 0.916366 | 1.000000 | No | No |
| log-sigma-1-0-mm-3D_glcm_ClusterProminence | 0.382 | 0.536409 | -0.009 | [-0.014, 0.074] | 0.916366 | 1.000000 | No | No |
| log-sigma-1-0-mm-3D_gldm_DependenceNonUniformity | 0.356 | 0.550802 | -0.009 | [-0.014, 0.075] | 0.921750 | 1.000000 | No | No |
| log-sigma-2-0-mm-3D_glcm_Autocorrelation | 0.356 | 0.550802 | -0.009 | [-0.014, 0.082] | 0.921750 | 1.000000 | No | No |
| log-sigma-1-0-mm-3D_glcm_ClusterShade | 0.343 | 0.558069 | -0.009 | [-0.014, 0.080] | 0.924478 | 1.000000 | No | No |
| log-sigma-2-0-mm-3D_glcm_ClusterProminence | 0.306 | 0.580145 | -0.010 | [-0.014, 0.087] | 0.929468 | 1.000000 | No | No |
| original_firstorder_Kurtosis | 0.306 | 0.580145 | -0.010 | [-0.014, 0.069] | 0.929468 | 1.000000 | No | No |
| original_shape_SurfaceVolumeRatio | 0.294 | 0.587594 | -0.010 | [-0.014, 0.068] | 0.929468 | 1.000000 | No | No |
| log-sigma-1-0-mm-3D_glcm_Correlation | 0.294 | 0.587594 | -0.010 | [-0.014, 0.076] | 0.929468 | 1.000000 | No | No |
| log-sigma-1-0-mm-3D_gldm_LargeDependenceLowGrayLevelEmphasis | 0.282 | 0.595086 | -0.010 | [-0.014, 0.079] | 0.929468 | 1.000000 | No | No |
| log-sigma-3-0-mm-3D_glcm_Autocorrelation | 0.282 | 0.595086 | -0.010 | [-0.014, 0.070] | 0.929468 | 1.000000 | No | No |
| log-sigma-1-0-mm-3D_glcm_JointEntropy | 0.260 | 0.610201 | -0.010 | [-0.014, 0.066] | 0.929566 | 1.000000 | No | No |
| original_shape_MajorAxisLength | 0.260 | 0.610201 | -0.010 | [-0.014, 0.066] | 0.929566 | 1.000000 | No | No |
| original_glcm_Correlation | 0.249 | 0.617822 | -0.010 | [-0.014, 0.083] | 0.929566 | 1.000000 | No | No |
| original_glcm_SumEntropy | 0.249 | 0.617822 | -0.010 | [-0.014, 0.070] | 0.929566 | 1.000000 | No | No |
| log-sigma-3-0-mm-3D_gldm_LargeDependenceEmphasis | 0.238 | 0.625484 | -0.011 | [-0.014, 0.076] | 0.932539 | 1.000000 | No | No |
| log-sigma-2-0-mm-3D_glszm_SmallAreaLowGrayLevelEmphasis | 0.188 | 0.664389 | -0.011 | [-0.014, 0.062] | 0.961894 | 1.000000 | No | No |
| log-sigma-2-0-mm-3D_gldm_SmallDependenceLowGrayLevelEmphasis | 0.188 | 0.664389 | -0.011 | [-0.014, 0.078] | 0.961894 | 1.000000 | No | No |
| original_glcm_ClusterProminence | 0.170 | 0.680216 | -0.011 | [-0.014, 0.065] | 0.961894 | 1.000000 | No | No |
| log-sigma-1-0-mm-3D_gldm_SmallDependenceHighGrayLevelEmphasis | 0.161 | 0.688184 | -0.012 | [-0.014, 0.068] | 0.961894 | 1.000000 | No | No |
| original_glszm_LowGrayLevelZoneEmphasis | 0.161 | 0.688184 | -0.012 | [-0.014, 0.066] | 0.961894 | 1.000000 | No | No |
| original_glszm_ZonePercentage | 0.161 | 0.688184 | -0.012 | [-0.014, 0.060] | 0.961894 | 1.000000 | No | No |
| log-sigma-3-0-mm-3D_glszm_LargeAreaHighGrayLevelEmphasis | 0.161 | 0.688184 | -0.012 | [-0.014, 0.071] | 0.961894 | 1.000000 | No | No |
| log-sigma-2-0-mm-3D_gldm_LargeDependenceHighGrayLevelEmphasis | 0.152 | 0.696185 | -0.012 | [-0.014, 0.075] | 0.961894 | 1.000000 | No | No |
| original_glszm_SizeZoneNonUniformityNormalized | 0.144 | 0.704221 | -0.012 | [-0.014, 0.068] | 0.961894 | 1.000000 | No | No |
| log-sigma-1-0-mm-3D_firstorder_90Percentile | 0.136 | 0.712290 | -0.012 | [-0.014, 0.067] | 0.961894 | 1.000000 | No | No |
| log-sigma-2-0-mm-3D_gldm_LargeDependenceEmphasis | 0.136 | 0.712290 | -0.012 | [-0.014, 0.073] | 0.961894 | 1.000000 | No | No |
| log-sigma-3-0-mm-3D_firstorder_Maximum | 0.128 | 0.720392 | -0.012 | [-0.014, 0.058] | 0.961894 | 1.000000 | No | No |
| original_shape_Elongation | 0.120 | 0.728525 | -0.012 | [-0.014, 0.068] | 0.961894 | 1.000000 | No | No |
| log-sigma-1-0-mm-3D_glszm_ZoneEntropy | 0.120 | 0.728525 | -0.012 | [-0.014, 0.067] | 0.961894 | 1.000000 | No | No |
| original_glszm_LargeAreaEmphasis | 0.113 | 0.736688 | -0.012 | [-0.014, 0.065] | 0.961894 | 1.000000 | No | No |
| log-sigma-1-0-mm-3D_glcm_Imc1 | 0.106 | 0.744882 | -0.012 | [-0.014, 0.058] | 0.961894 | 1.000000 | No | No |
| log-sigma-1-0-mm-3D_glrlm_RunEntropy | 0.106 | 0.744882 | -0.012 | [-0.014, 0.054] | 0.961894 | 1.000000 | No | No |
| log-sigma-2-0-mm-3D_firstorder_Maximum | 0.099 | 0.753104 | -0.013 | [-0.014, 0.070] | 0.964914 | 1.000000 | No | No |
| log-sigma-3-0-mm-3D_ngtdm_Contrast | 0.068 | 0.794620 | -0.013 | [-0.014, 0.058] | 0.982773 | 1.000000 | No | No |
| log-sigma-1-0-mm-3D_glszm_SmallAreaLowGrayLevelEmphasis | 0.073 | 0.786266 | -0.013 | [-0.014, 0.063] | 0.982773 | 1.000000 | No | No |
| log-sigma-3-0-mm-3D_glcm_JointEntropy | 0.062 | 0.802997 | -0.013 | [-0.014, 0.064] | 0.982773 | 1.000000 | No | No |
| log-sigma-2-0-mm-3D_firstorder_Skewness | 0.062 | 0.802997 | -0.013 | [-0.014, 0.065] | 0.982773 | 1.000000 | No | No |
| original_glszm_LargeAreaHighGrayLevelEmphasis | 0.062 | 0.802997 | -0.013 | [-0.014, 0.066] | 0.982773 | 1.000000 | No | No |
| log-sigma-1-0-mm-3D_glrlm_RunLengthNonUniformity | 0.062 | 0.802997 | -0.013 | [-0.014, 0.060] | 0.982773 | 1.000000 | No | No |
| log-sigma-1-0-mm-3D_firstorder_Kurtosis | 0.052 | 0.819820 | -0.013 | [-0.014, 0.058] | 0.991298 | 1.000000 | No | No |
| original_shape_LeastAxisLength | 0.043 | 0.836725 | -0.013 | [-0.014, 0.067] | 0.991298 | 1.000000 | No | No |
| log-sigma-2-0-mm-3D_glszm_LargeAreaHighGrayLevelEmphasis | 0.038 | 0.845207 | -0.013 | [-0.014, 0.061] | 0.991298 | 1.000000 | No | No |
| log-sigma-3-0-mm-3D_ngtdm_Strength | 0.038 | 0.845207 | -0.013 | [-0.014, 0.061] | 0.991298 | 1.000000 | No | No |
| log-sigma-3-0-mm-3D_glszm_LargeAreaLowGrayLevelEmphasis | 0.030 | 0.862224 | -0.013 | [-0.014, 0.060] | 0.991298 | 1.000000 | No | No |
| original_glcm_MaximumProbability | 0.026 | 0.870756 | -0.013 | [-0.014, 0.062] | 0.991298 | 1.000000 | No | No |
| log-sigma-3-0-mm-3D_gldm_LargeDependenceLowGrayLevelEmphasis | 0.023 | 0.879304 | -0.014 | [-0.014, 0.058] | 0.991298 | 1.000000 | No | No |
| log-sigma-1-0-mm-3D_ngtdm_Complexity | 0.023 | 0.879304 | -0.014 | [-0.014, 0.055] | 0.991298 | 1.000000 | No | No |
| log-sigma-2-0-mm-3D_glcm_ClusterTendency | 0.014 | 0.905028 | -0.014 | [-0.014, 0.058] | 0.991298 | 1.000000 | No | No |
| log-sigma-2-0-mm-3D_glrlm_LongRunLowGrayLevelEmphasis | 0.012 | 0.913627 | -0.014 | [-0.014, 0.061] | 0.991298 | 1.000000 | No | No |
| log-sigma-2-0-mm-3D_glszm_LowGrayLevelZoneEmphasis | 0.014 | 0.905028 | -0.014 | [-0.014, 0.059] | 0.991298 | 1.000000 | No | No |
| log-sigma-2-0-mm-3D_glcm_ClusterShade | 0.014 | 0.905028 | -0.014 | [-0.014, 0.056] | 0.991298 | 1.000000 | No | No |
| log-sigma-3-0-mm-3D_gldm_SmallDependenceLowGrayLevelEmphasis | 0.017 | 0.896441 | -0.014 | [-0.014, 0.050] | 0.991298 | 1.000000 | No | No |
| original_shape_VoxelVolume | 0.009 | 0.922235 | -0.014 | [-0.014, 0.054] | 0.991298 | 1.000000 | No | No |
| log-sigma-1-0-mm-3D_gldm_DependenceEntropy | 0.004 | 0.948111 | -0.014 | [-0.014, 0.051] | 0.991346 | 1.000000 | No | No |
| original_firstorder_Minimum | 0.007 | 0.930853 | -0.014 | [-0.014, 0.056] | 0.991298 | 1.000000 | No | No |
| original_gldm_LargeDependenceEmphasis | 0.009 | 0.922235 | -0.014 | [-0.014, 0.057] | 0.991298 | 1.000000 | No | No |
| original_glszm_SizeZoneNonUniformity | 0.009 | 0.922235 | -0.014 | [-0.014, 0.050] | 0.991298 | 1.000000 | No | No |
| original_glszm_ZoneEntropy | 0.009 | 0.922235 | -0.014 | [-0.014, 0.059] | 0.991298 | 1.000000 | No | No |
| log-sigma-2-0-mm-3D_glszm_SmallAreaEmphasis | 0.009 | 0.922235 | -0.014 | [-0.014, 0.053] | 0.991298 | 1.000000 | No | No |
| log-sigma-2-0-mm-3D_glszm_SizeZoneNonUniformity | 0.007 | 0.930853 | -0.014 | [-0.014, 0.056] | 0.991298 | 1.000000 | No | No |
| log-sigma-3-0-mm-3D_glcm_MaximumProbability | 0.001 | 0.974042 | -0.014 | [-0.014, 0.057] | 0.991346 | 1.000000 | No | No |
| log-sigma-2-0-mm-3D_glszm_ZoneEntropy | 0.001 | 0.974042 | -0.014 | [-0.014, 0.054] | 0.991346 | 1.000000 | No | No |
| log-sigma-3-0-mm-3D_glszm_ZoneEntropy | 0.002 | 0.965394 | -0.014 | [-0.014, 0.059] | 0.991346 | 1.000000 | No | No |
| log-sigma-1-0-mm-3D_glcm_Imc2 | 0.002 | 0.965394 | -0.014 | [-0.014, 0.060] | 0.991346 | 1.000000 | No | No |
| log-sigma-3-0-mm-3D_glrlm_LongRunLowGrayLevelEmphasis | 0.002 | 0.965394 | -0.014 | [-0.014, 0.055] | 0.991346 | 1.000000 | No | No |
| log-sigma-2-0-mm-3D_gldm_LargeDependenceLowGrayLevelEmphasis | 0.001 | 0.982693 | -0.014 | [-0.014, 0.059] | 0.991346 | 1.000000 | No | No |
| original_gldm_DependenceEntropy | 0.001 | 0.982693 | -0.014 | [-0.014, 0.050] | 0.991346 | 1.000000 | No | No |
| log-sigma-3-0-mm-3D_glszm_GrayLevelNonUniformityNormalized | 0.000 | 0.991346 | -0.014 | [-0.014, 0.056] | 0.991346 | 1.000000 | No | No |
| original_gldm_SmallDependenceLowGrayLevelEmphasis | 0.000 | 0.991346 | -0.014 | [-0.014, 0.053] | 0.991346 | 1.000000 | No | No |

Notes: 164 features retained after Spearman correlation clustering (|r| ≥ 0.90) from 386 initially extracted features. Test: Kruskal-Wallis H (non-parametric). Effect size: η² with 95% bootstrap confidence interval (1,000 iterations). Corrections: Benjamini-Hochberg FDR (primary) and Bonferroni (secondary), both at α = 0.05. Features with η² < 0 reflect numerical artefacts from the bootstrap estimator at very small effect sizes and should be interpreted as η² ≈ 0.

Supplementary Table S7 — MRI Acquisition Sensitivity Analysis

Purpose: To assess whether the primary radiomic classification result was driven by the three examinations acquired with minority MRI protocols.

| **Analysis cohort** | **n (high/low)** | **Balanced accuracy (95% CI)** | **AUC (95% CI)** | **.632+ AUC** | **Permutation p** |
| --- | --- | --- | --- | --- | --- |
| Full cohort | 74 (40/34) | 0.69 (0.58–0.80) | 0.73 (0.61–0.84) | 0.71 | 0.001 |
| Dominant protocol only | 71 (37/34) | 0.67 (0.56–0.78) | 0.68 (0.55–0.81) | 0.68 | 0.003 |

Notes: The dominant-protocol sensitivity analysis excluded T1W_SPIR hals SENSE, t1_se_tra_gd and T1W gd CLEAR (one case each) and reran the entire locked primary pipeline. All three minority-protocol cases were in the high-risk stratum; protocol group was not significantly associated with risk stratum (two-sided Fisher exact p = 0.245). Confidence intervals were obtained by bootstrap resampling. Permutation p values are based on 1,000 whole-pipeline label shuffles with +1 smoothing. Because only seven examinations were acquired at 3 T, field-strength-specific estimates and ComBat-type harmonisation were not considered reliable.

**Supplementary Figures**

**Supplementary Figure S1. Held-out validation of automated tumour segmentation.**

(A) Case-level volumetric Dice similarity coefficient (DSC) for the 20 manually annotated NFPA cases used in Phase 1 held-out cross-validation. Each case was evaluated exactly once by a model not trained on that case. The solid blue vertical line indicates median DSC; dashed vertical lines indicate DSC thresholds of 0.70, 0.80, and 0.90. Orange points indicate low-DSC cases (DSC < 0.60); blue points, DSC ≥ 0.60. (B) Predicted versus ground-truth tumour volume for the same 20 held-out cases. The dashed grey line indicates perfect agreement between predicted and ground-truth volume. Pearson correlation is shown for volumetric agreement. Segmentation performance was assessed only in the annotated validation cohort and does not represent independent external validation. DSC, Dice similarity coefficient; NFPA, non-functioning pituitary adenoma.Alt text: Dice score plot and predicted-versus-reference volume plot summarising automated tumour segmentation performance in held-out validation cases.
